# Trust Dynamics and Verbal Assurances in Human Robot Physical Collaboration

**DOI:** 10.3389/frai.2021.703504

**Published:** 2021-07-20

**Authors:** Basel Alhaji, Michael Prilla, Andreas Rausch

**Affiliations:** ^1^ Simulation Science Center Clausthal-Göttingen, Clausthal University of Technology, Clausthal-Zellerfeld, Germany; ^2^ Institute for Informatics, Clausthal University of Technology, Clausthal-Zellerfeld, Germany; ^3^ Institute for Software and System Engineering, Clausthal University of Technology, Clausthal-Zellerfeld, Germany

**Keywords:** human-robot collaboration, trust dynamics, trust factors, trust calibration, human robot teamwork, verbal feedback

## Abstract

Trust is the foundation of successful human collaboration. This has also been found to be true for human-robot collaboration, where trust has also influence on over- and under-reliance issues. Correspondingly, the study of trust in robots is usually concerned with the detection of the current level of the human collaborator trust, aiming at keeping it within certain limits to avoid undesired consequences, which is known as trust calibration. However, while there is intensive research on human-robot trust, there is a lack of knowledge about the factors that affect it in synchronous and co-located teamwork. Particularly, there is hardly any knowledge about how these factors impact the dynamics of trust during the collaboration. These factors along with trust evolvement characteristics are prerequisites for a computational model that allows robots to adapt their behavior dynamically based on the current human trust level, which in turn is needed to enable a dynamic and spontaneous cooperation. To address this, we conducted a two-phase lab experiment in a mixed-reality environment, in which thirty-two participants collaborated with a virtual CoBot on disassembling traction batteries in a recycling context. In the first phase, we explored the (dynamics of) relevant trust factors during physical human-robot collaboration. In the second phase, we investigated the impact of robot’s reliability and feedback on human trust in robots. Results manifest stronger trust dynamics while dissipating than while accumulating and highlight different relevant factors as more interactions occur. Besides, the factors that show relevance as trust accumulates differ from those appear as trust dissipates. We detected four factors while trust accumulates (perceived reliability, perceived dependability, perceived predictability, and faith) which do not appear while it dissipates. This points to an interesting conclusion that depending on the stage of the collaboration and the direction of trust evolvement, different factors might shape trust. Further, the robot’s feedback accuracy has a conditional effect on trust depending on the robot’s reliability level. It preserves human trust when a failure is expected but does not affect it when the robot works reliably. This provides a hint to designers on when assurances are necessary and when they are redundant.

## Introduction

The exponential growth of machine’s and robot’s intelligence made it possible for robots and autonomous systems to work physically alongside humans, interacting and collaborating with them and supporting them in many domains. This dramatic advent of technology opens up many opportunities to support human work and to create new forms of technology-supported collaborative work. It shifts the robots and other intelligent system’s roles from being perceived and used as tools into being perceived as teammates (Groom and Nass, 2007) that can augment the abilities of humans and allow for hybrid team formation. This new kind of teamwork has the potential to collaboratively achieve more than any single entity of its members can achieve on its own. It can increase the team performance and reduce the human workload. However, many challenges accompany this development. For example, it might reduce the human sense of autonomy (Blake, 2020; Tan and Taeihagh, 2020) by forcing people to adhere to what the machine needs. Additionally, it is mostly challenging for humans to comprehend the limits of intelligent systems designed by others, which puts them in an uncertain environment, especially when no experience exists with the machine (Wagner and Robinette, 2021). Therefore, in order for this kind of teams to succeed and be beneficial for humans, the collaboration between the team members needs to be carefully designed. In this work, we are looking into how humans and robots can collaborate together autonomously on both sides.

A key factor that strongly influences the quality of this collaboration is human trust due to its influences on over- and under-reliance issues in this form of teamwork (Lee and See, 2004). Inappropriate reliance problems in human-robot collaboration often come in conjunction with inappropriate trust the human has toward the robotic team partner (Parasuraman and Riley, 1997; Hoff and Bashir, 2015). Therefore, trust must be kept within the borders of proper reliance especially in the case of physical collaboration as human safety could be at stake. This process is widely known as trust calibration (Atkinson and Clark, 2013; de Visser et al., 2017). Having an accurate model of human trust in autonomous robots is a prerequisite for trust calibration as it can be used by the robot to estimate trust of the human in it and adjust its behavior in accordance.

Human trust toward a robot partner, however, is a latent variable that has shown to be sensible to several factors (Hancock et al., 2011; Hoff and Bashir, 2015), which makes its modeling a challenge as the model should include most factors. On the other hand, trust is known to be a context-dependent construct (Yagoda and Gillan, 2012). Hence, factors that lead human trust in a given context X might not necessarily play an active role it in another context Y. Therefore, human trust toward a robot should be modeled given the collaboration context. The majority of the prior research to identify trust factors does not consider the new emerging context of human robot physical collaboration in industrial setting (Charalambous et al., 2016), as traditionally robots are separated from humans e.g., by the means of physical safety fences. In order to model trust in such a context, we need first to investigate the relevant factors.

Additionally, trust is known to have dynamics. It develops over time (increases or decreases) as more and more interactions occur (Jonker et al., 2004; Lee and See, 2004). We believe that trust dynamics and how its factors affect it dynamically are essential cornerstones for its modeling. Existing methods to measuring trust commonly depend on post-hoc questionnaires since trust is not directly observable. These questionnaires are usually administered at the end of an experiment. Measuring trust at the end of an experiment, however, fails to provide information about how it evolves, let alone the dynamic effect of the factors, which requires deeper analysis.

Further, researchers suggested that providing assurances (e.g., explanations and confidence levels) from the robot or other forms of artificially intelligent agents to the human during the execution of a given task has the potential to calibrate human trust (see Israelsen and Ahmed, 2019 for a survey).

We believe that in such a hybrid team formulation that emulates an all-human team a higher-level communication could be beneficial such as using natural language.

In this work, we aim at finding relevant trust factors in a physical collaboration setting as well as trust own dynamic behavior. In addition to that, we study the effect of the robot’s verbal feedback and test whether it forms a means to calibrate human trust during the collaboration.

## Background

There exists no universally accepted definition of trust in the current literature. It has multiple definitions across different disciplines. In psychology for example, trust between humans has been defined as “*a psychological state comprising the intention to accept vulnerability based upon positive expectations of the intentions or behavior of another*” (Rousseau et al., 1998). This definition is based on the definition out forward by Mayer et al. stating that trust is “*the willingness of a party to be vulnerable to the actions of another party based on the expectation that the other will perform a particular action important to the trustor, irrespective of the ability to monitor or control that other party*” (Mayer et al., 1995).

In human-automation interaction literature, a widely used definition of trust has been given by Lee and See who defined trust of a human in a machine as “*the attitude that an agent will help achieve an individual’s goals in a situation characterized by uncertainty and vulnerability*” (Lee and See, 2004), which we adopt in this work. It can be noticed that most definitions of trust in the literature are centered around an agent (human) being *vulnerable* and *uncertain* about the outcome of an interaction with another agent (in our case, a robot). Thus, trust is of major importance in situations that include these two attributes.

In general, human trust has two bases: cognition-base and affect-base (Johnson and Grayson, 2005). Cognition-based trust is known to be knowledge-based that depends on rational judgement of the competence and dependability of the trustee. Affect-based trust on the other hand is more emotional-based that includes the feeling or confidence that the trustee is protective and concerned for the welfare of the trustor (Bente et al., 2008). The involvement of emotions in trust building and the subjectivity of the concept makes its modeling a challenging task.

### Modeling Trust and its Factors

Trust is being studied extensively in many different (not necessarily related) research domains, where researchers are attempting to develop accurate models that explain the concept and identify the factors and dimensions that shape it. For example, in interpersonal trust literature, trust has been modeled by three different dimensions in a hierarchal stage manner by Rempel et al. (1985) where it dynamically develops over time. According to this model, trust at any stage depends on the outcome of the other earlier stages. The components of trust model in this work are predictability, dependability, and faith. These three components form the stages of the model, and they occur in the aforementioned order. This model has been tested by Muir in the context of human-automation interaction (Muir, 1994). The result indicates that the model is also valid for this context. Accordingly, trust in automation as well is not a simple variable but rather a complex multidisciplinary context dependent construct that consists of many different dimensions (Yagoda and Gillan, 2012). This also applies for many other fields where trust has an influence on the whole interaction outcome [e.g., in technology (Gulati et al., 2017), in computers (Nothdurft et al., 2014), in automation (Lee and See, 2004; Hoff and Bashir, 2015; John D.), and in robots (Hancock et al., 2011; Yagoda and Gillan, 2012)].

Models and factors identified in a given field do not necessarily transfer to another because of the differences between the contexts and the way the interaction takes place. For example, in human-technology/computer interaction context, Gulati et al. studied the role of trust looking for attributes of technical artifacts that directly affect human trust in them (Gulati et al., 2018). They found that willingness of the user to interact with a technical artifact, perceived competence and benevolence associated with a technical artifact, and reciprocity are the main attributes that affect human trust in artifacts. The used artifact was Siri in this study, which does not have a physical embodiment. Robots, in contrast to other intelligent artifacts, possess physical existence attributes and are usually designed in a way that emulates other living creatures (e.g., animals, insects and humans). For human trust in automation context, Hoff and Bashir proposed a detailed model that consists of three layers: dispositional, situational, and learned trust (Hoff and Bashir, 2015). Each layer of the model encompasses different factors that play a role in shaping it. From this work, one can notice that trust is influenced by numerous factors.

This fact also holds true in the context of human-robot trust. In this regard, a meta-analysis over the existing literature has been conducted by Hancock et al. (2011). The identified factors that affect trust have been clustered into three categories: human-related (ability-based and characteristics), robot-related (performance-based and attribute-based), and environmental (team collaboration and tasking). The result of the analysis shows that robot-related performance-based factors have the strongest association with human trust (Hancock et al., 2011). These factors include dependability, reliability, predictability among others. Specifically, the reliability of the robot (in both objective and subjective sense) seems to be one of the major factors that affect human trust because it severely impacts the perceived performance. We use the term *robot objective reliability* (*reliability* henceforward) as being able to do the assigned job successfully. Failing to do so has a strong negative impact on human trust which has been demonstrated by several research work (Desai et al., 2013; Hancock et al., 2011; Salem et al., 2015; Ye et al., 2019). *Perceived reliability* refers to the sysem consistency in operation from the user’s perspective (Larasati et al., 2020), which has also shown to be an important factor for trust development (Charalambous et al., 2016; Madsen and Gregor, 2000).

An additional literature survey on human trust in robots by Law and Scheutz (2021) also divided trust into two categories: performance-based trust and relational-based trust. Performance-based trust refers to the case where the robot does not interact with people but separated in place. The relational-based trust, however, is more about social activity (e.g., nursing). Nevertheless, in the context of human-robot collaboration, even in industrial settings, these two notions of trust cannot be separated, since collaborating with a robot as a team partner already embraces many social aspects.

Robot’s attributes also appear to play a role in shaping human trust. Natarajan et al. studied human’s trust in a robotic teammate as a function of different robot attributes in a decision support scenario (Natarajan and Gombolay, 2020). The considered attributes are anthropomorphism, robot presence, and type of provided support. The results indicate that trust and anthropomorphism are positively correlated, whereas the physical presence of the robot did not prove to have significant influence on human trust.

The collaboration setting where a human and a robot collaborate physically in industry is relatively new. Most of the prior work, based on which the current trust factors are identified, do not consider physical human robot collaboration. Hence, there is a lack of knowledge about trust factors in physical collaboration settings, which calls for further investigations. Human-robot collaboration developers who aim to account for human trust during the interaction need to first identify the relevant trust factors in the given collaboration setting, because of trust context dependency.

### Trust Dynamics

In addition to being affected by many factors, human trust is known to be a dynamic phenomenon that changes over time as many research works suggest (Jonker et al., 2004; Lee and See, 2004; Rempel et al., 1985). Thus, it is of high importance to understand trust evolvement in a dynamic manner. The majority of trust researchers, though, resort to post-hoc questionnaires as the main strategy to estimate and model trust since it is a latent variable and it is still challenging to measure it. This, however, typically provides a measurement of trust at a single point of time (Guo and Yang, 2020) usually administered at the end of an experiment.

To study human trust dynamically, Hu et al. conducted experiments on a computer-based interface where participants reported their trust in a machine in multiple trials to capture its development (Hu et al., 2019). The machine (a simple simulation of an autonomous driving car) in these experiments provides the user with the sensor output and leaves the decision to the human to trust it or not. The authors were able to identify trust as a linear dynamical system with high accuracy. In this work, trust is mainly modeled as a function of experience which in turn is operationalized to be a function of misses and false alarms and there was no direct collaboration between the user and the system.

Xu and Dudek adopted a “performance-centric” view of trust and developed an online probabilistic trust model called OPTIMo (Xu and Dudek, 2015). The model deploys a Dynamic Bayesian Network (DBN), where human trust is represented as a belief state that can be inferred from observations. The DBN is trained with the data collected from participants who supervised a boundary-tracking robot in a simulated environment and were prompted to report their trust periodically.

Other researchers also modeled trust as a function of performance and implemented a computational model of dynamic trust as a deterministic time-series [originally identified by Lee and Moray (1992)] with different measures of performance depending on the task (Rahman et al., 2015; Walker et al., 2015; Rahman et al., 2016). In these works, physical collaboration takes place in assembly and hand-over tasks.

Most of the models that consider the dynamics of trust are computational which can be helpful to make the robot aware of its partner/user trust level. Although these models have proved to enhance the overall interaction and collaboration, they strongly simplify the concept of trust and do not consider all trust factors relevant to the use cases. Additionally, even the performance-based view of trust includes many other factors as Hancock et al. (2011) illustrate, such as predictability and adaptability which are overlooked in the proposed models. Moreover, the difference between the dynamics of trust in accumulation and dissipation directions is still understudied.

In order for the model to capture human trust, we first need deeper understanding on its dynamics in both directions and to know what factors affect its evolution and how exactly. It is yet unknown, whether the statically identified factors in the literature remain relevant in dynamic models.

### Trust Calibration

In addition to its modeling and measuring, trust calibration is a challenging and critical task as well. It is actually the goal of most of trust researchers in the human-robot interaction field. A collaborative robot that is considered as a team partner should make sure that the human partner does not deploy it to tasks that it was not designed for or overly trust its skills in situations which are unfamiliar to the robot (Alhaji et al., 2020). The robot should as well (at least try to) dissuade the human partner from placing him-/herself or the overall task at risk (Wagner et al., 2018). Finding and designing methods and means that can be used by the robot to maintain human trust in it and repair it when needed is then particularly critical, knowing that trust is easy to lose and difficult to gain (Juvina et al., 2019).

Because of their contribution to human trust evolvement, Tolmeijer et al. recently developed a taxonomy that investigates the types of failure in human-robot interaction as the main cause for trust violation and studied their potential impact on human trust and repair (Tolmeijer et al., 2020). The categories of failure they proposed are: design failure (when the system is not ideal in the real human-robot interaction setting), system failure (when the system acts different than intended), expectation failure (when system actions differ from human expectations), and user failure (which can be caused by the other categories). The authors also proposed strategies to deal with each category of failures. However, how trust is affected as a function to these failures was not considered.

Israelsen and Ahmed’s survey focuses on means of trust calibration, which are some programmed components of an artificially intelligent agent (e.g., robot) that are engineered to address the user trust (Israelsen and Ahmed, 2019). They refer to these methods as assurances and classify them into *hard* and *soft assurances*. Hard assurances offer formal guarantees that the system works according to predefined specifications which are usually necessary for certification. Soft assurances, on the other hand, are more user-centered and meant to adjust user’s trust. This work highlights the importance of the assurances that an agent should provide. It shows a one-way trust cycle that exists between a human and an artificially intelligent agent (e.g., robot) in which the robot perceives the trust-related behavior of the human and provides assurances in order to affect human’s trust.

One possible soft assurance that can be used by the robot is providing information to the human about the actual abilities and limitations the robot has. Desai et al. (2013) have studied the impact of failure and feedback on trust in a teleoperation task. They tested two types of feedback (sematic and non-sematic) from the robot to the human to indicate the robot’s own confidence about its sensor data. They found that this confidence feedback improves the control allocation strategy without altering trust levels.

Verbal feedback has shown to be a means to improve human robot collaboration performance (St. Clair and Mataric, 2015), because a team of people relies heavily on verbal communication to succeed. With the increasing intelligence and perception abilities of robots, a hybrid team of humans and robots might take advantage of such an anthropomorphic communication way. The question, however, remains whether such kind of feedback can be used as a means for trust maintenance and as means to avoid inappropriate deployment. In the coming sections, we use the words assurance and feedback interchangeably.

### Open Issues and Research Questions

As demonstrated above, trust has a dynamic nature. It changes over time as a function of changing experience (Jonker and Treur, 1999), and so do the factors that influence it. Hence, in order to appropriately quantify trust, its dynamics must be taken into consideration. However, most of the existing methods on modeling and measuring trust rely mainly on post-hoc questionnaires at the end of an experiment which provides only a “snapshot view” of trust (Guo and Yang, 2020) instead of measuring it continuously.

Although a couple of models already exist that aim to capture trust dynamically, trust dynamics itself is still vague in the current literature (see *Trust Dynamics*). In addition to that, to have better understanding on dynamic human trust development in robots, we need to identify the factors that play a role in a given collaboration context and we need to understand how the relation between these factors and trust changes dynamically over time, because they have to be incorporated in any trust model. However, the factors that are dynamically related to trust as it accumulates and as it dissipates, which are essential components for trust modeling, are still hardly known. This forms a major gap that we are going to address in this work.

Besides, human-robot collaboration in close proximity and hybrid team formation is an emerging interaction setting. While there is intensive research work on human-robot trust as shown in *Modeling Trust and its Factors*, there is a lack of knowledge about the factors of trust in a synchronous and co-located teamwork setting let alone its dynamics in such a case (*Trust Dynamics*), where the human and the robot execute interdependent actions and share the same workspace. Accordingly, the research questions that guide this part of the work regarding the dynamics of trust are:

• RQ1: How does human trust in a robotic partner dynamically evolve over time in a physical collaboration setting?
• RQ2: What factors are dynamically related to human trust in accumulation and in dissipation in human-robot physical collaboration?

Further, calibration of trust is as critical as its modeling. It is of equal importance to find means that can be used by the robot to influence human trust in it (Desai et al., 2013). Especially, when the collaboration environment includes risk. Assurances from the robot side are meant to tune user’s trust. The most intuitive and natural way for non-expert users to receive and share information is using natural languages (Mavridis, 2015), and we know from the literature that verbal feedback has a positive influence on team performance and enhances the perception of the robot as a teammate (see *Trust Calibration*), but the influence of this kind of feedback on human trust when the robot verbally conveys its abilities and limitations in the given collaboration setting is currently absent in the literature and requires further investigation. Correspondingly, the following research questions are addressed in this work:

• RQ3: What effects do verbal assurances from the robot have on human trust in it?
• RQ4: When should the robot provide assurances in order to maintain human trust?

## Methodology

In order to examine the dynamics of trust as well as the impact of verbal feedback on it, we conducted a two-phase experiment where participants had to collaborate with a collaborative robot (aka. CoBot) to disassemble a simplified model of an electric car traction battery. In the first phase, we studied the dynamics of trust in a within-subject design by manipulating the reliability of the robot in two different runs (reliable/unreliable) without any feedback. For the second phase, we used a 2x2 mixed-design in which we manipulated both reliability and feedback. In this phase, feedback correctness was manipulated within-subject (correct/incorrect) and reliability was manipulated between-subject (reliable/unreliable). For this study, we simulated the Panda CoBot from Franka Emika^1^, which is a typical industrial robot with 7 degrees of freedom in a mixed-reality environment. Thirty-two participants took part in this experiment. Half of them identified as women and the other half identified as men. They were aged between eighteen and forty-one years. Participants were recruited using an internal university participant pool, word of mouth, and the available university channels and forums. All of them participated voluntarily. Twenty-nine participants have no experience with autonomous machines and none of them have experience with robotic manipulators. Only five participants used the HoloLens before during experiments for less than 2 h. One whole trial in our study lasted for 50–60 min and each participant was compensated with 10 euro for participation. Each participant has witnessed four different conditions which we detail in the coming sections.

### Disassembly Scenario

In the research project HerMes^2^, we aim at designing human-machine and human-robot hybrid teams that support a circular economy. In this case, the robot plays the muscles by taking over the repetitive and burdening tasks from the human partner, and the human brings flexibility and high perception capabilities to the team. The combination of the different but complementary abilities of both humans and robots allows for the prospect of harnessing the strength of them both in different applications.

Disassembly is one of the many applications in which full automation is not feasible and this combination can be a successful solution. Other applications include search and rescue, military, and space (Goodrich and Schultz, 2007). Disassembly is an essential step of the End-of-Life (EoL) process of used products, which cannot be considered as reversed assembly (Vongbunyong and Chen, 2015). For humans, the disassembly tasks can be very burdensome. However, most of the disassembly tasks in disassembly factories^3^ are currently conducted manually by human workers (Chang et al., 2017). The reason behind this is that the environments of this kind of factories are very dynamic in nature because of the uncertainties that are associated with the unpredictable characteristics of the products at their EoL stage of their lifecycle, which makes the full automation of the disassembly processes not possible at present.

The scenario of our experiment is in the same vein. It emulates the process of the disassembly of electric car traction batteries in a disassembly factory (like Umicore^4^). This process involves high risk because of the hazardous substances that are contained in the cells of a battery (Wegener et al., 2015). Any damage to the cells during the disassembly can threaten the human health. Consequently, it is essential to have a well calibrated trust for a successful collaboration between humans and robots as teams in these environments.

The disassembly process of traction batteries differs from model to model and the steps vary in accordance. In our work, we concentrate on the first step of the *general* process of disassembling an electric car battery, which is: *opening the battery system by removing the cover* (Wegener et al., 2015). In this step, the robot is supposed to support the human in loosening the screws that hold the cover of the battery. If the human does not rely on the robot to do this task for him/her, s/he should perform this task manually, which will prevent a potential higher performance and increases the human workload. On the other hand, if the human overly relies on the robot, s/he might expect it to safely detect and loosens screws with special abnormal conditions. The screws in the battery can be in countless different conditions at the end of the life cycle of a battery. They could be broken, dirty, rusty, or even missing, which substantially increases the challenge of automatic recognition of the screws and their real condition. It is also unknown whether the robot would be able to handle them since currently robots cannot be designed to deal with all uncountable possible variations in the environment. This would be very risky without the human intervening. This initial step of a battery disassembly already shows how important it is to have an appropriate level of reliance in this form of collaboration.

### Environment Choice

As mentioned earlier, our experiment takes place in a mixed reality environment. We chose mixed reality because of the risky scenario we are using. We wanted to make this risk of the scenario clear to the participants without actually posturing them to real danger with a real robot.

Mixed reality is a special form of virtual reality. Whereas virtual reality completely separates the user from the real world, mixed reality merges the real and the virtual world by overlaying virtual objects in the real world allowing the user to interact with both virtual and real objects. Several research studies used virtual reality to simulate a robot in human-robot interaction studies [e.g., (Fratczak et al., 2019; Müller et al., 2017; van den Brule et al., 2014; Weistroffer et al., 2013; Zhu et al., 2020) just to name a few]. Although this might not supplant a real-world experiment, it allows for ad-hoc prototyping of real robotic systems for different applications. This is because of the flexibility this technology provides to researchers allowing them to avoid the overhead associated with handling technical and hardware issues (e.g., in our case autonomous detection of battery and screws positions). Additionally, previous research has found that using virtual reality can be a valid option to study human-robot interaction in manipulation scenarios (Duguleana et al., 2011). This makes the use of mixed reality a plausible option for an initial step toward our goals.

### Tasks

**FIGURE 1:**
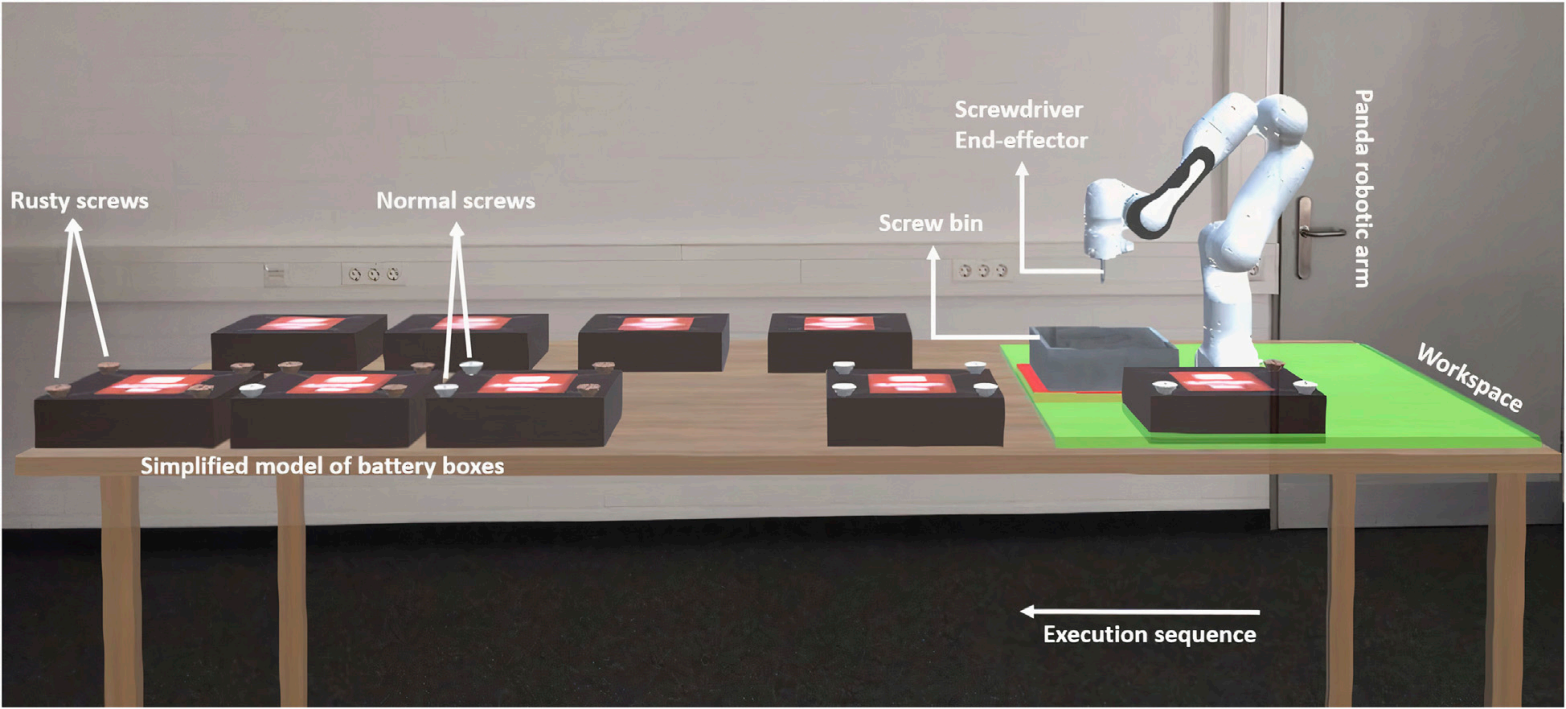
Experiment setup (from participant’s point of view). The green area shows the accessible workspace of the robot. There are two rows of batteries: on the front side, the batteries with screws are located; on the back side, some extra already disassembled batteries are located to create a more realistic scene.

In our study, we set the goal of the human in this collaboration setting to be loosening as many screws as possible with the help of the robot. The robot has a screwdriver as an end-effector and can autonomously detect the position of the battery and its screws. The task of the human is to bring a battery and place it somewhere accessible by the robot (inside the workspace) whereas the robot task is to loosen the screws for the human. This process is repeated until no batteries with screws are left. There were five batteries in total with four screws each. The number of screws having a special condition increases gradually from having only normal screws in the first battery to four abnormal screws for the last one. In our experiment, we chose the rusty condition as an abnormal one. The human has to handle the batteries in their order starting from the closest to the robot base, which does not have any rusty screws, and going outward. The human has the freedom to choose what screw the robot should loosen. Figure 1 illustrates the experimental setup.

### Experimental Conditions

As illustrated in the related work (see *Modeling Trust and its Factors*), the ability of the robot to execute its assigned task correctly (Reliability) is one of the major factors that affect human’s trust dynamically. Additionally, assurances and warnings (Feedback) from the robot side have the potential to successfully calibrate the trust of the human in it. Therefore, in this experiment, we manipulated these two variables. Each one of them has two different levels explained in the following:

Reliability: this variable has been used to excite trust of the human in the positive and negative directions, which allows us to observe the human’s trust evolvement. The levels of this variable are:

• Reliable behavior: in the reliable case, the robot successfully loosens all rusty and normal screws and brings them to the screw bin correctly (see Figure 1). This level is presumed to excite trust in the positive direction.
• Unreliable behavior: in this case, the robot loosens the normal screws correctly but fails to loosen the rusty ones in different ways. Examples include getting stuck (second battery), failing to bring the screws to the bin (third battery), getting stuck again (fourth battery), and failing to detect the position of the screws in the first place (fifth battery). In this case, the robot hits the battery in the middle which causes an explosion. This level is presumed to excite trust in the negative direction.

These two conditions address both research questions RQ1 and RQ2, in which we are interested in exploring human trust dynamics and the factors associated with its evolvement.

Feedback: this variable is used to check the impact of accurate and inaccurate assurances/warnings on the trust of the human. The feedback was only provided if the screw is rusty. This variable has two levels as well, which are intended to calibrate human trust during the operation. These levels are:

• Correct feedback: in this case the robot verbally provides the human with assurances/warnings about its ability to loosen a specific screw. For example, in the case of reliable behavior, the robot will say “*I Can*” as an assurance to the human that the robot is actually able to loosen the screw. In the case of unreliable behavior, the robot says “*I Cannot*” as a warning.
• Incorrect feedback: similar to the correct feedback case, in this case the robot verbally provides the human with assurances/warnings about its ability to loosen a specific screw. Incorrectly though. For example, in the case of reliable behavior, the robot will say “*I Cannot*” as a warning to the human that the robot is actually not able to loosen the screw. In the case of unreliable behavior, the robot says “*I Can*” as a misleading assurance.

These two conditions address both research questions RQ3 and RQ4, in which we are interested in exploring the effect of verbal feedback on human trust and whether it forms a means for trust calibration.

### Apparatus

**FIGURE 2:**
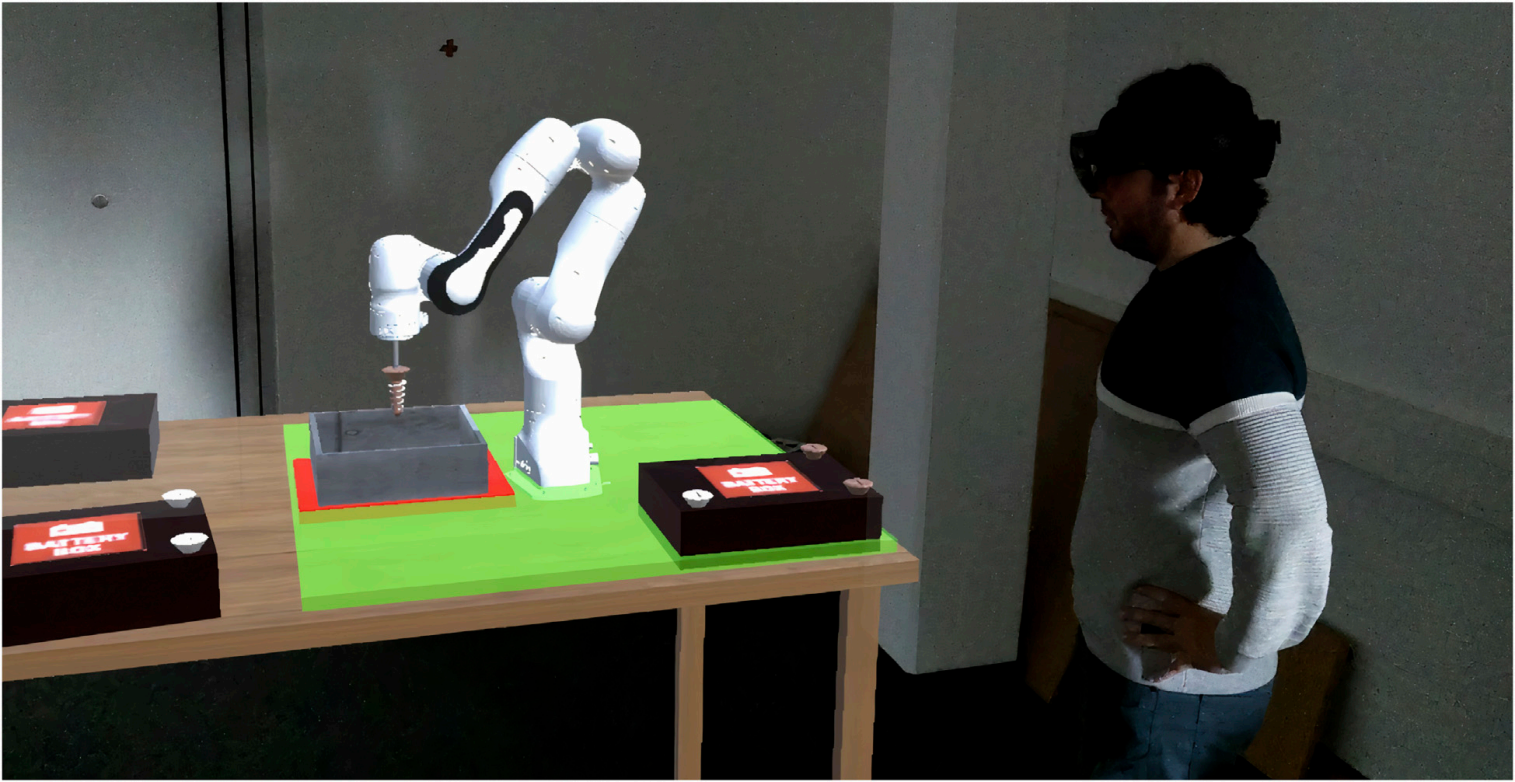
Third party point of view. A participant wears the HoloLens and commands the robot to loosen screws. It is allowed to locate the battery anywhere inside the green workspace except over the screw bin which is marked in red.

Our experiment took place in a mixed-reality environment using a Microsoft HoloLens 2 (HL2)^5^, which is a head-mounted display that allows for 3D visualization of and interaction with virtual objects (see Figure 2). It has a wide field of view (52° diagonal) with 47 pixel per degree resolution. It can be controlled naturally by finger gestures and voice commands. We designed our software using Unity^6^ and C#.

### Experimental Procedure

Upon arrival, participants were asked to fill in a demographic questionnaire. Afterward, participants put on the HoloLens and started a training session in which they familiarized themselves with the CoBot and with the environment. They also trained on how to pick up a battery and bring it to the correct location (the green area in Figure 2). The experimenter provided an overview about how the experiment will run and explained what tasks the robot performs and what tasks the participant has to perform. Next, participants removed the HoloLens to read the scenario in which they get more information about the environment, the abilities of the robot, and the interactions available. We told the participants, in written form and orally, that it is guaranteed that the robot is able to automatically recognize and loosen screws if they are in normal condition, whereas, as with many robotic devices, this behavior cannot be assured for the vast number of different situations the robot may encounter. Therefore, its behavior with abnormal screws (rusty in our case) is uncertain in nature. We mentioned this to create an atmosphere of uncertainty about the outcome of the collaboration. In addition to that, we told them that if the robot mistakenly misses a screw and hits the body of the battery with the screwdriver end-effector, an explosion might occur which can be a life-threatening event. This shall make the human vulnerable in the collaboration with the vulnerability being in the form of a physical hazard.

Afterward, participants were assigned to one of two main groups. We will refer to the first group as *G1* and the second one as *G2*. Different research work suggests that there is a difference between men and women in terms of trust [e.g., trust games in economics (Dittrich, 2015), human robot interaction (Gallimore et al., 2019), and human automation interaction (Schuster et al., 2015)]. Therefore, we made sure that the number of men and women was equal in the groups. Both groups started with the reliability manipulation phase, which we call *Trust Dynamics (TD),* and afterward they got to the *Trust Calibration (TC)* phase where only the feedback accuracy changes and reliability is held constant. Table 1 illustrates the groups and the conditions they experienced.

**TABLE 1:** Experiment groups and conditions. Reliable: succeeds with all screws. Unreliable: succeeds with normal screws but fails with rusty ones. Correct Feedback: when the screw is rusty, the robot says “I Can” if it will succeed and “I Cannot” if it will fail. Incorrect Feedback: when the screw is rusty, the robot says “I Cannot” if it will succeed and “I Can” if it will fail.

	Trust dynamics (Within-subject)	Trust calibration (2x2 Mixed-Design)	
Group G1 (*n* = 16)	Reliable (REL)	Reliable + Correct Feedback (REL+CF)	
	Unreliable (UNREL)	Reliable + Incorrect Feedback (REL+IF)	
Group G2 (*n* = 16)	Reliable (REL)		Unreliable + Correct Feedback (UNREL+CF)
	Unreliable (UNREL)		Unreliable + Incorrect Feedback (UNREL+IF)

**FIGURE 3:**
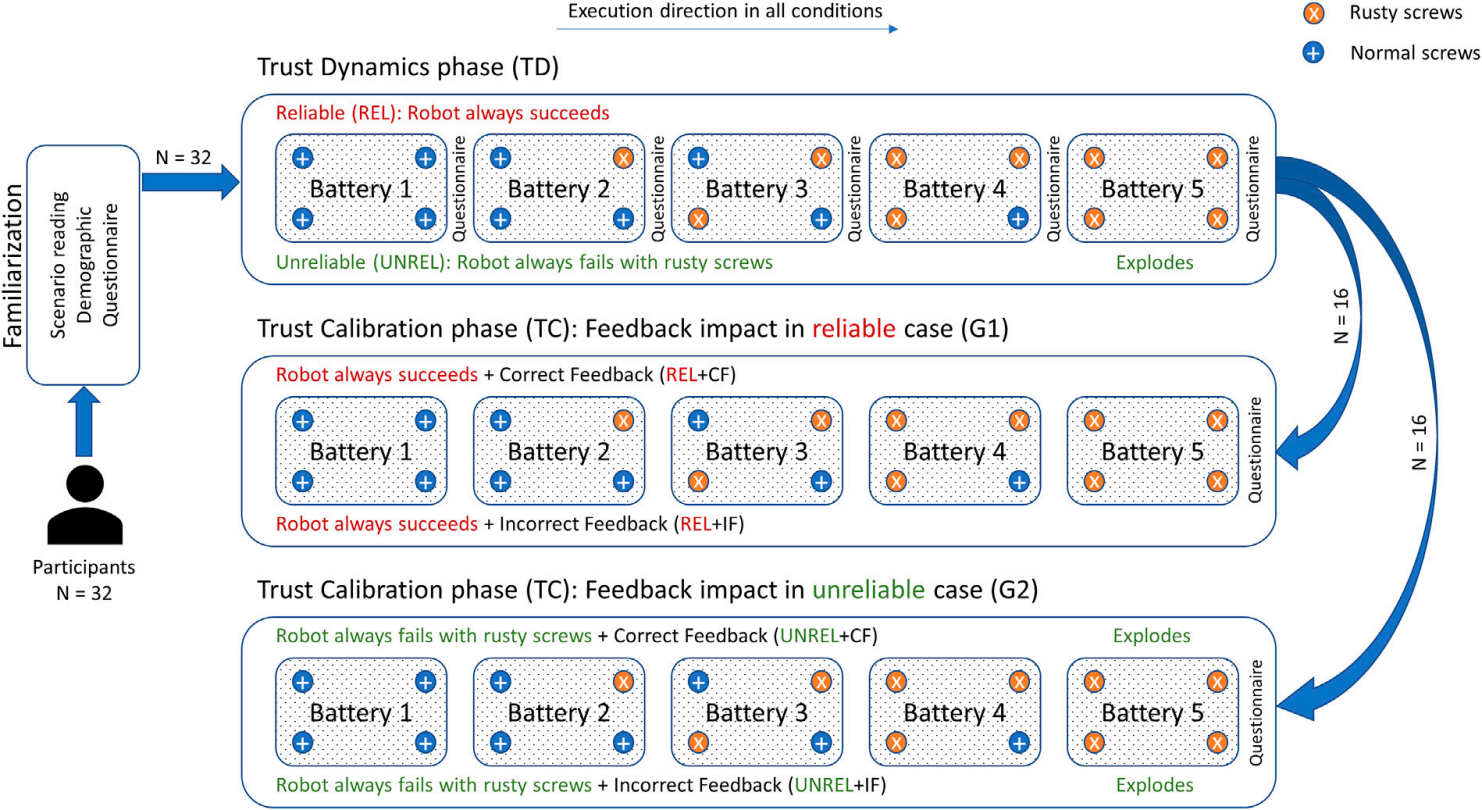
Experimental procedure. Participants start with TD phase and fill a questionnaire after each battery. They proceed with TC phase depending on the group, where only one questionnaire is administered. Batteries should be handled in their order. In TD phase, trust dynamics is studied. In the TC phase, the effect of verbal feedback on human trust is studied with different reliability levels.

In the Trust Dynamics phase (TD), participants experienced reliable and unreliable behavior of the robot in two separate runs, which shall excite human trust in the positive and negative directions, respectively. The runs were fully counterbalanced; thus, half of the participants witnessed the reliable behavior first and then witnessed the unreliable behavior. The other half has experienced exactly the opposite sequence. For the purpose of understanding how trust evolves over time, participants in this phase have filled out a questionnaire after each battery (five times in total), so we get multiple snapshots of trust as it changes after each interaction to keep track of its accumulation or dissipation. In the Trust Calibration phase (TC), the robot acts always reliably in G1 and the correctness of the feedback it gives changes between two separate runs. In contrast to G1, the robot in G2 acts always unreliably and the correctness of the given feedback changes in two separate runs. In the two parts of the TC phase, we are interested in exploring the effect of correct/incorrect verbal feedback on trust in reliable/unreliable behavior and whether it can be used as a means to calibrate human trust. To measure this effect, we asked participants to fill out a questionnaire at the end of each run. The order of the runs was also fully counterbalanced between participants. Figure 3 depicts the whole experiment procedure.

The experiment ends with a short debriefing session in which we asked the participants about their experiences and personal opinions with regard to the different conditions they went through.

#### Verbal Commands

In this experiment, the human and the robot interact verbally. The recognizable set of commands the human can use is as follows:

• Ready: Used when the human has already positioned the battery correctly. Here, the robot responds with “*OK*” to acknowledge the reception of the command.
• One, Two, Three, and Four: Used to indicate which screw the human wants the robot to loosen. One command can be used at a time. For example, if human says “*One*” to loosen the first screw, he should wait until the robot is done with the screw before using the next command, “*Four*” for example.
• Stop: Forces the robot to go back to its initial configuration and abort the task at hand. This was to be used when the robot fails to continue a certain task.
• Go: After the robot gives its feedback to the human whether it can or cannot loosen the desired screw, it waits until the human approves the execution of the task using this command. Although some participants believed the feedback given by the robot and were hesitant about approving the execution especially when the robot says “*I Cannot*”, in our experiment, the human has no other option than approving, because we need to see the impact of the feedback variable.

### Measures

**FIGURE 4:**
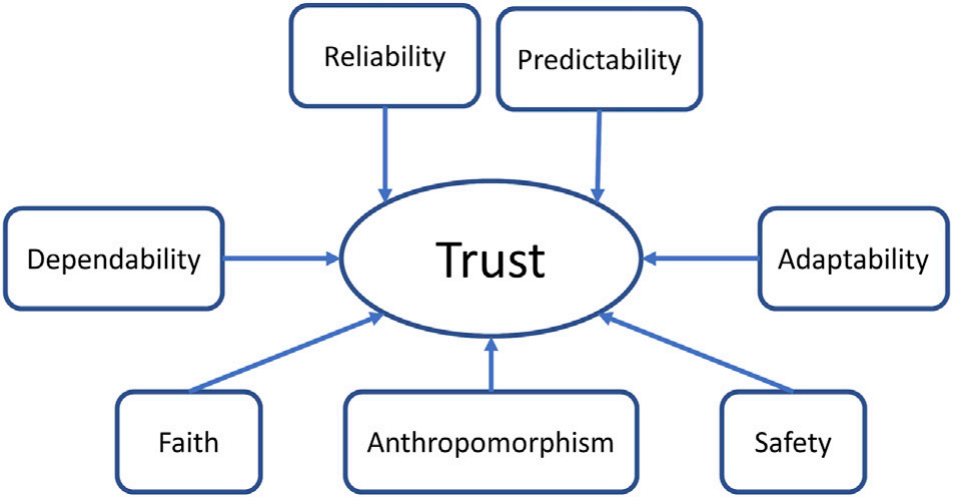
Potential trust factors included in this study.

Similar to multiple previous research (Madhani et al., 2002; Brown and Galster, 2004; Hergeth et al., 2015; Hergeth et al., 2016), we used a single item to measure trust and each factor that might have an influence on it. We used the factors collected in the robot-related category of Hancock et al. (2011) study and Muir and Moray (1996) as potentially relevant factors to dynamically affect trust in our setting (see *Modeling Trust and its Factors*). We concentrate mainly on factors that have a dynamic nature. These factors are: Dependability, Reliability, Predictability, Adaptability, Anthropomorphism, Safety/Proximity, and Faith as shown in Figure 4. We included faith since it seems to be an important factor from the studies of Rempel et al. (1985) and Muir (1994). We excluded the factors that do not fit the scenario or the collaboration setting we are using. For example, we excluded the level of automation factor since in our design this is a static one.

To measure these variables, we selected items from different questionnaires of prior studies that measure the factors we are interested in. The items and the factors they intend to measure are listed below:

• Perceived Dependability: we used the item from Muir questionnaire *to what extent can you count on Panda to do its job?* [Muir 1989; as cited in Desai (2012)].
• Perceived Reliability: we used the item from Charalambous questionnaire *the robot did not seem reliable* (Charalambous et al., 2016).
• Perceived Predictability: we used the item from Muir questionnaire *to what extent can the robot’s behavior be predicted?* [Muir 1989; as cited in Desai (2012)].
• Perceived safety: we used the item from Garza questionnaire *during the experiment I felt unsafe when the robot was physically close to me* (Garza, 2018).
• Perceived Adaptability: we used the item from Conti *the robot adapts its behavior according to my preferences* (Conti et al., 2019).
• Anthropomorphism: we used two items of the Godspeed questionnaire which ask participants to rate their impression of the robot on the scales of *machine-like* and *human-like* as well as on the scale of *unconscious* to *conscious* (Bartneck et al., 2009).
• Faith: we used the item from Muir questionnaire *to what extent do you believe that the robot will be able to cope with all situations in the future?* [Muir 1989; as cited in Desai (2012)].
• For trust itself, we used also the item from Muir questionnaire *overall how much do you trust the robot?* [Muir 1989; as cited in Desai (2012)].

The selection process of these items is based on how such variables are usually measured in the literature. We selected the items that best fit our experimental setting from previously developed questionnaires. We used seven points Likert-type scale to measure the factors ranging from Strongly Disagree/Not at All to Strongly Agree/To a Great Extent. The questionnaire was administered in both German and English languages and the items were translated into German by a native speaker.

### Statistical Analysis

Our data are ordinal in nature as they are collected by Likert-type scales. For this kind of data, non-parametric tests are suitable (Field and Hole, 2003). Thus, we employ two-tailed *Wilcoxon Signed-Rank* and *Mann-Whitney U* tests to make pairwise comparisons for dependent and independent samples respectively (within and between groups). The significance level used in this work is α= 0.05 for all statistical tests adjusted with Bonferroni correction in the case of multiple pairwise comparisons. We performed all statistical tests and graphics representations using libraries in Python (SciPy: Virtanen et al., 2020; NumPy: Harris et al., 2020; Pandas: McKinney, 2010; Matplotlib: Hunter, 2007).

## Results

### Reliability Manipulation (TD)

In this phase as shown in Table 1, thirty-two participants witnessed the reliable and unreliable behavior of the robot (fully counterbalanced) without any feedback. We will refer to the condition when the robot always acts reliably as *REL*, and to the case when the robot always acts unreliably as *UNREL*. Half of the participants in this phase (16 participants) started with the REL condition and then moved to the UNREL condition. We refer to this sequence as *REL*→*UNREL*. The other half did exactly the opposite sequence which we will refer to as *UNREL*→*REL* (see Figure 3).

#### Sequence Impact

As mentioned in *Trust Calibration*, trust cannot be easily repaired. It takes a long time for the human to recover from a trust violation. Therefore, as the order of our experimental conditions (reliable REL, unreliable UNREL) are completely counterbalanced, we need to check whether it is feasible to combine the data collected from REL condition in the sequence REL→UNREL with REL condition in the sequence UNREL→REL and the same for UNREL condition data. For this purpose, we compare REL last results of REL→UNREL sequence with REL last results of UNREL→REL sequence. Accordingly, we use Mann-Whitney U test as we are comparing two separate groups in this case.

Results reveal that the resulting trust in the REL condition is higher for the sequence REL→UNREL (M = 6.12, SD = 0.88) than trust in the reverse sequence UNREL→REL (M = 5.88, SD = 0.96). However, this difference is not significant (*n* = 16, U = 148, *p* = 0.426). Similarly, trust in the UNREL condition of the sequence REL→UNREL (M = 3.06, SD = 1.48) is also higher than in the sequence UNREL→REL (M = 2.31, SD = 1.35), but not significantly neither (*n* = 16, U = 166, *p* = 0.141). Finally, we also compare the level of trust in the first step of the REL condition in the sequence REL→UNREL (M = 5.62, SD = 0.88) with the first step of the REL condition in the sequence UNREL→REL (M = 4.81, SD = 1.76) and similarly we perform a comparison for the UNREL condition for both sequences REL→UNREL (M = 6.0, SD = 0.97) and UNREL→REL (M = 5.75, SD = 1.0). The assumption we are checking here is that priming participants with reliable/unreliable behavior considerably impacts their propensity to trust the robot in the next run, which might cause a trust repair problem that needs to be considered. Mann-Whitney U test did not show statistical significance neither in the REL (*n* = 16, U = 161.5, *p* = 0.192) nor in the UNREL (*n* = 16, U = 147.0, *p* = 0.451) conditions. Therefore, we did not have any trust repair problems caused by the different sequences and from now on we will ignore the sequence.

#### Reliability Effect

The reliability of the robot’s behavior is probably the most important characteristic that it should have in order to gain human trust (see *Modeling Trust and its Factors*). We presume that reliable behavior has a positive effect on human trust whereas unreliable behavior is presumed to affect it negatively as current state of the art indicates. To make sure that in our experiment the change in the robot’s behavior did excite trust in the intended direction, we compare the last step of the REL (M = 6.0, SD = 0.92) with the last step of the UNREL (M = 2.69, SD = 1.45) conditions (see Figure 3). Here we want to check whether witnessing the robot always succeed in loosening the rusty screws is associated with a higher trust as compared to the case where it always fails. Results of Wilcoxon signed-rank test show statistically significant difference between the reliable and unreliable conditions (*n* = 32, W = 0, *p* = 0.000*). Thus, this variable does have a significant impact on human trust and can be used as intended.

#### Gender Effect

Previous work suggests differences between men and women in terms of trust. Therefore, we check whether we can observe similar differences in our experiment. However, the results of Mann-Whitney U test show no statistical differences between men and women in all five steps of both reliable and unreliable behavior as illustrated in Table 2.

**TABLE 2:** Results of men vs. women trust comparisons over the five steps. No statistically significant difference has been detected.

Steps	Men (*n* = 16)		Women (*n* = 16)		Statistics (U)	*p*
	M	SD	M	SD		
**REL**						
1	5.38	1.54	5.06	1.34	149	0.413
2	5.82	1.38	5.44	1.26	157	0.251
3	5.88	1.20	5.56	1.03	154.5	0.297
4	6.06	1.12	5.56	1.15	163.5	0.162
5	6.25	0.77	5.75	1.0	164	0.152
**UNREL**						
1	6.06	0.85	5.69	1.08	153.5	0.312
2	5.44	1.31	5.0	1.21	153.5	0.318
3	4.94	1.06	4.56	1.46	143	0.557
4	4.31	1.74	4.44	1.46	121	0.787
5	2.94	1.48	2.44	1.41	151.5	0.362

#### Trust Dynamic Evolution

Since the sequence does not seem to have a significant impact on the development of trust, we study the dynamics of trust as it accumulates and as it dissipates regardless of the sequence participants went through. Hence, the sample size for this part is *n* = 32.

##### Trust Accumulation (Reliable)

**FIGURE 5:**
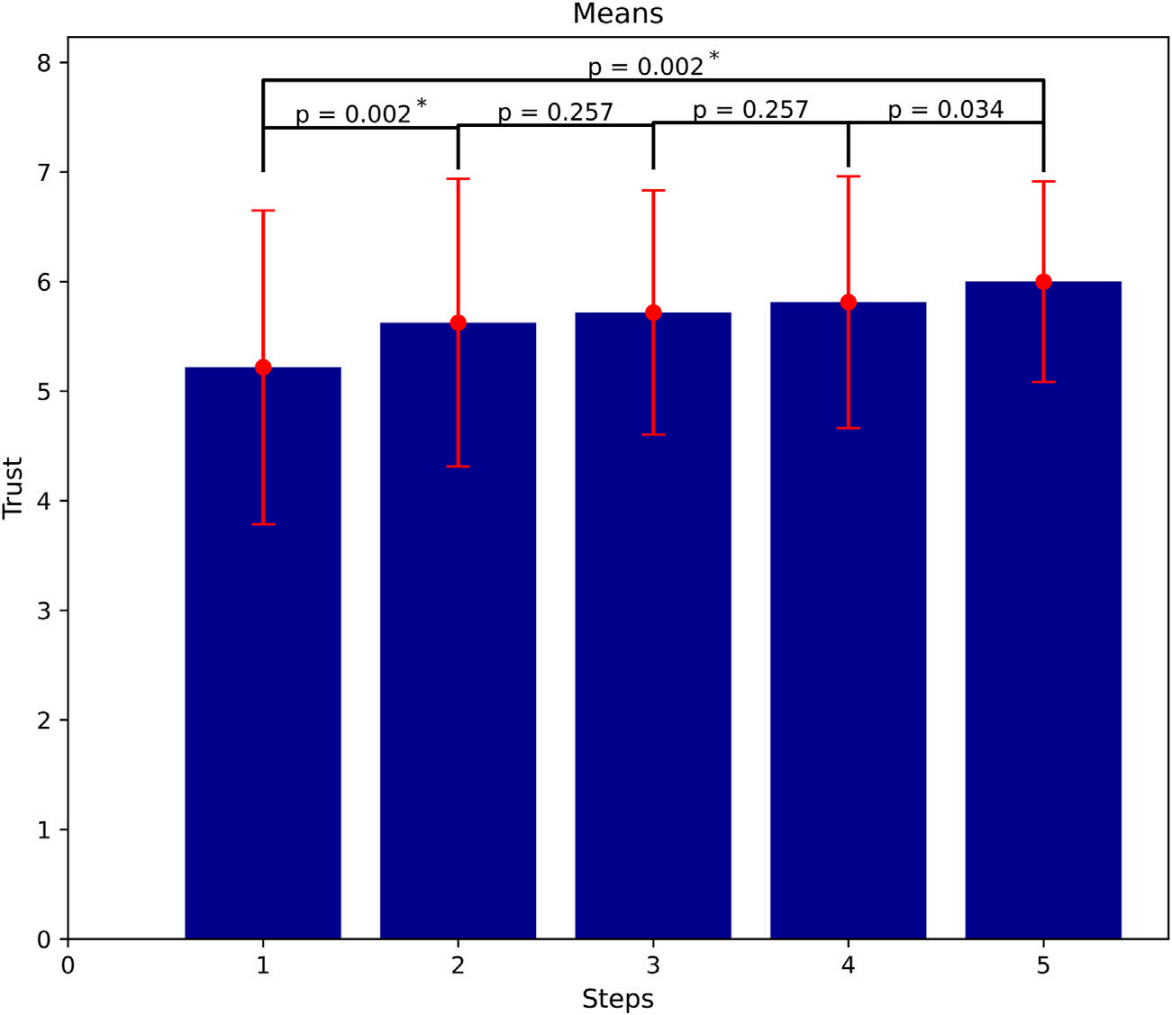
Trust accumulation as more successful interactions and task executions occur (means of trust at all steps). Significant gain of trust between the first and the last steps, but not in between them ( α=0.01 ).

We pair-wisely compare the reported trust after each battery in the REL condition using Wilcoxon signed-rank test. Because we make five comparisons here, we use Bonferroni correction to calculate our corrected significance level, which is $\alpha_{corrected}=\frac{\alpha }{5}=\;0.01$ . In the case where the robot acts always reliably, human trust shows a statistically significant development over the five steps. However, it does not show a statistically significant development in between the steps except between the first and the second ones. Figure 5 illustrates the trust accumulation process, where it starts from a relatively high value (M = 5.22, SD = 1.43) after the first battery and increases after successful handling of the rusty screws.

##### Trust Dissipation (Unreliable)

**FIGURE 6:**
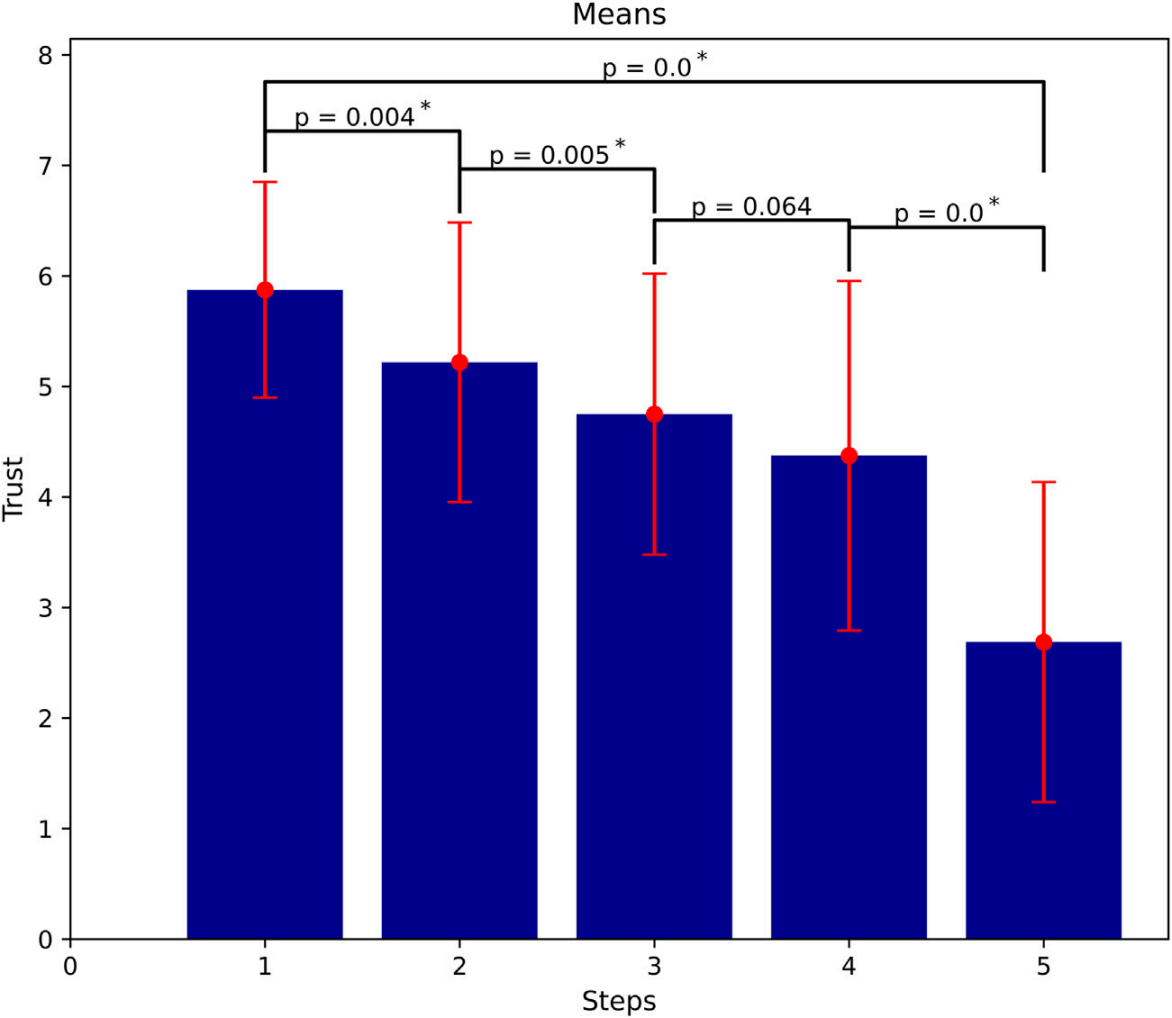
Trust dissipation as more failures occur (means of trust at all steps). Significant loss of trust mostly even in between steps ( α=0.01 ).

Similar to the reliable case, we compare the reported trust after each battery in the UNREL condition using pair-wize Wilcoxon signed-rank test. The results show that, when the robot acts always unreliably, trust does show statistically significant development over the five different steps similar to the accumulation case ( $\alpha_{corrected}=0.01$ ). The difference, however, is that in the negative direction there mostly is significant development in between the steps which implies a stronger dynamic. Trust in this case as well starts from a relatively high value (M = 5.88, SD = 0.98) after the first battery and decreases strongly after each failure. Figure 6 shows the dissipation process of trust.

##### Trust Dynamics

**FIGURE 7:**
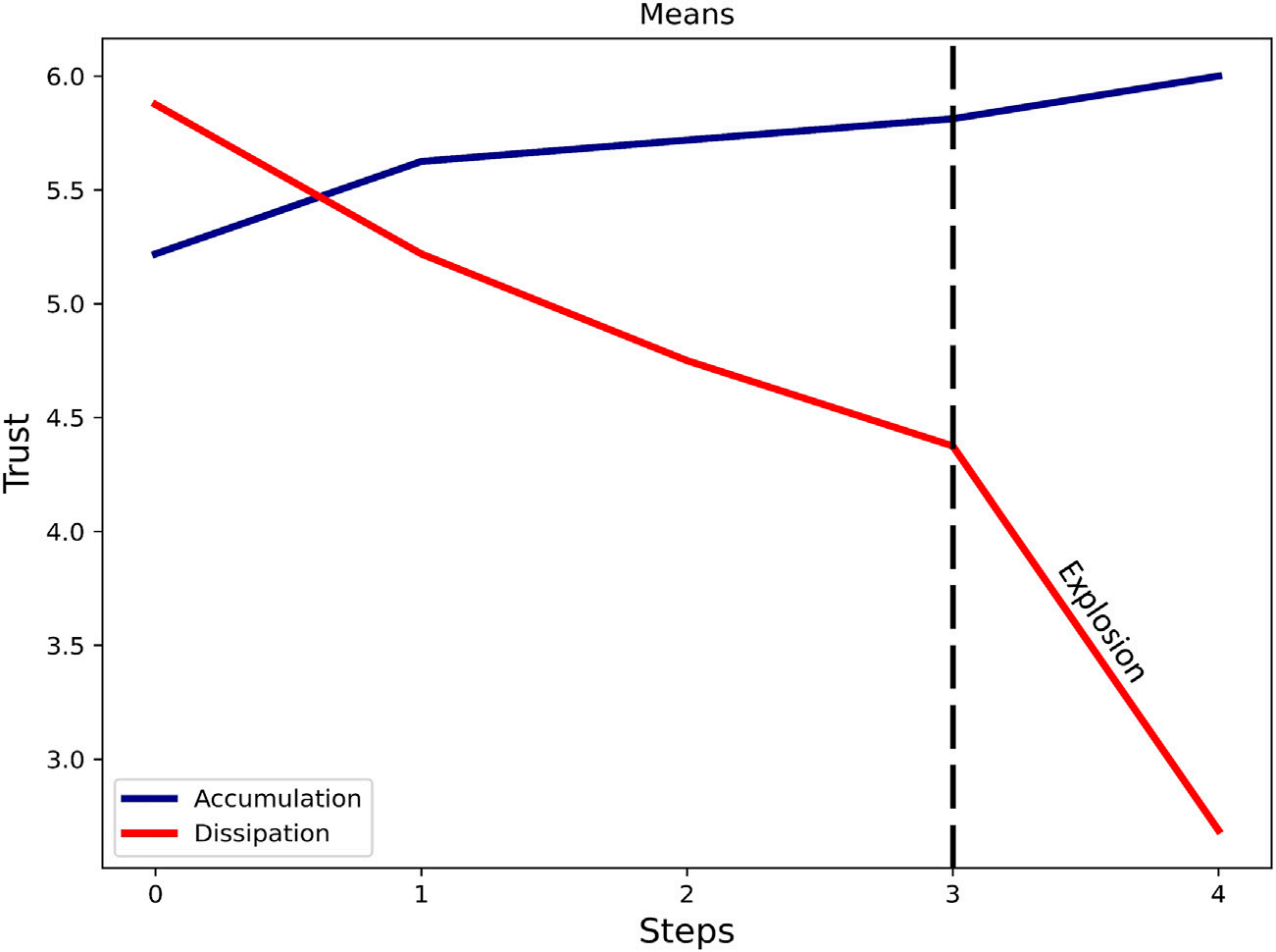
Dynamic trust development. Almost doubly stronger dynamics in dissipation compared to accumulation.

Figure 7 shows how trust accumulates and dissipates in our experiment. It can be seen that the slope of the trust accumulation line is almost half the slope of trust dissipation one even when the explosion part is excluded (see *Experimental Conditions*), which means that its dynamics is stronger as it dissipates than its dynamics as it accumulates. It also empirically shows evidence that trust is difficult to build and easy to lose.

##### Trust Factors in Accumulation

In order to find the factors that affect trust in this collaboration scenario, we conduct Spearman correlation analysis to find out what factors go along with trust as it increases. It is common in the literature to consider a correlation to be high when the correlation coefficient is equal or greater than 0.5 (Bröhl et al., 2019) or 0.7 (Ratner, 2009). In our study, we decided to use a middle ground and we will consider a correlation as high if the correlation coefficient is equal to or greater than 0.6 ( $r_{s}\;\geq 0.6$ ). The result of this analysis is illustrated in Table 3.

**TABLE 3:** Factors strongly correlated with trust in accumulation phase over the five steps (Spearman $r_{s}=0.6,\alpha =0.05$ ). Multiple factors show consistent correlation with trust.

Steps	Factors	Correlation	*p*-value*
1	dependability	0.6343	0.0001
	predictability	0.65	0.0001
	faith	0.784	0.000
2	dependability	0.6733	0.000
	reliability	0.6271	0.0001
	predictability	0.66	0.000
	adaptability	0.6175	0.0002
	faith	0.887	0.000
3	dependability	0.6882	0.000
	reliability	0.7433	0.000
	predictability	0.7073	0.000
	adaptability	0.6192	0.0002
	faith	0.8663	0.000
4	dependability	0.65	0.0001
	reliability	0.6401	0.0001
	predictability	0.6561	0.000
	safety	0.634	0.0001
	adaptability	0.7059	0.000
	faith	0.7939	0.000
5	dependability	0.6113	0.0002
	reliability	0.6287	0.0001
	predictability	0.6346	0.0001
	faith	0.7884	0.000

The first step’s result shows three factors that strongly correlate with trust. They are predictability, dependability, and faith. From the second step onward, reliability joins the other factors and continues to strongly correlate with trust afterward. One can also notice that the safety factor appears only once which we attribute to the use of a virtual robot instead of a real one which in turn has increased participants perception of safety. Additionally, adaptability appears a couple of times starting from the second step and then disappears in the final step. This inconsistency could be due to the high subjectivity of the measured data, as participants sometimes attributed adaptability to the robot while the behavior of the robot was constant. Since both factors do not show consistency, we will not consider them as relevant factors in this work.

As a result, based on our correlational analysis, it seems like trust is dynamically associated with four factors that affect its accumulation as shown in Table 3. This needs further investigation to validate whether a causal relationship exists between the identified factors and human trust.

##### Trust Factors in Dissipation

We follow the same process by conducting Spearman correlation analysis and analyze the factors that associate trust as it decreases. A similar correlation criterion is used here ( $r_{s}\;\geq 0.6$ ). Table 4 contains the result of this analysis.

**TABLE 4:** Factors strongly correlated with trust in dissipation phase over the five steps ( $r_{s}=0.6,\alpha =0.05$ ). No consistent correlation has been detected.

Steps	Factors	Correlation	*p*-value*
1	safety	0.7459	0.000
2	reliability	0.6714	0.000
	predictability	0.6459	0.0001
3	-	-	-
4	adaptability	0.6468	0.0001
5	adaptability	0.7195	0.000
	faith	0.7149	0.000

In this case, none of the considered factors show a coherent relation to trust. Only adaptability appears twice at the end of the run. Similar to the accumulation, reliability also appears in the second step but disappears afterward.

### Reliable Behavior With Feedback Correctness Changing (TC in G1)

In this condition (see Table 1), we study the effect of assurances from the robot side on the human trust and whether they can be used as means to calibrate it. There are multiple forms of feedback the robot can provide to the human collaborator about its confidence and abilities (see *Trust Calibration*). In our experiment, we used a verbal form of assurances. In this condition, we study the impact of correct and incorrect verbal feedback on trust in the reliable behavior condition.

To analyze the effect of feedback accuracy, we compare reliable behavior of the robot (REL), where no feedback was provided, with reliable behavior with correct feedback (REL+CF) and incorrect feedback (REL+IF) respectively. The REL condition, however, has five different data points (one after each battery, see Figure 3). Therefore, for a fair comparison, we use the data from the last step of the REL condition which is equivalent to the data of REL+CF and REL+IF (see Figure 3). For this, we apply Wilcoxon signed-rank test with a Bonferroni-corrected significance level of α= 0.025 . The result shows no statistically significant difference (*n* = 16, W = 25.5, *p* = 0.477) between the two conditions REL_last_ (M = 5.81, SD = 0.83) and REL+CF (M = 5.62, SD = 0.81). Thus, correct feedback did not increase human trust when the behavior is reliable. Similarly, when incorrect feedback was provided, Wilcoxon signed-rank test shows a statistically significant difference (*n* = 16, W = 13, *p* = 0.021*) between the two conditions REL_last_ (M = 5.81, SD = 0.83) and REL+IF (M = 4.5, SD = 1.55). Hence, incorrect feedback did decrease human trust when it accompanies reliable behavior.

Although men reported higher trust than women in both REL+CF (men: M = 5.88, SD = 0.64; women: M = 5.38, SD = 0.92) and REL+IF (men: M = 5.0, SD = 1.60; women: M = 4.0, SD = 1.41) conditions, these differences did not show statistical significance in any of them (REL+CF: *n* = 8, U = 43.5, *p* = 0.194; REL+IF: *n* = 8, U = 43.5, *p* = 0.212).

### Unreliable Behavior With Feedback Correctness Changing (TC in G2)

In this condition, we want to test the impact of correct and incorrect feedback when the robot acts always unreliably. We compare the data from the last step of the unreliable behavior of the robot (UNREL) with unreliable behavior with correct feedback (UNREL+CF) and incorrect feedback (UNREL+IF), respectively. The result of Wilcoxon signed-rank test with Bonferroni correction ( α=0.025 ) shows a statistically significant difference between the two conditions UNREL_last_ (M = 3.25, SD = 1.53) and UNREL+CF (M = 4.88, SD = 1.20), thus correct feedback appears to have a positive impact on trust when the behavior is unreliable (*n* = 16, W = 6, *p* = 0.015*). However, Wilcoxon signed-rank test shows no statistically significant difference (*n* = 16, W = 16.5, *p* = 0.074) between the two conditions UNREL_last_ (M = 3.25, SD = 1.53) and UNREL+IF (M = 4.19, SD = 1.17). Hence, incorrect feedback did not seem to influence human trust when the robot is unreliable.

In this condition as well, men reported higher trust than women in the UNREL+CF (men: M = 5.0, SD = 1.51; women: M = 4.75, SD = 0.89) and in the UNREL+IF (men: M = 4.62, SD = 1.06; women: M = 3.75, SD = 1.16) conditions. However, we did not observe a statistical significance (UNREL+CF: *n* = 8, U = 33, *p* = 0.913; UNREL+IF: *n* = 8, U = 45, *p* = 0.159).

### Further Feedback Accuracy Effect (TC in G1 and G2)

To further explore the effect of feedback on human trust, we check the impact of correct feedback with both behavioral levels (reliable/unreliable), and the impact of incorrect feedback as well. This comparison takes place between the groups G1 and G2 (see Table 1).

First, we compare the reliable with correct feedback condition (REL+CF) with the unreliable with correct feedback condition (UNREL+CF). We apply Mann-Whitney U test here because we have two independent samples (see Table 1). The result shows that the trust level in the REL+CF (M = 5.62, SD = 0.81) is statistically significantly higher (*n* = 16, U = 179, *p* = 0.045*) than its level in the UNREL+CF condition (M = 4.88, SD = 1.20). This means the correct feedback effect on human trust is dominated by the effect of the unreliable behavior of the robot.

Similarly, we compare the reliable with incorrect feedback condition (REL+IF) with the unreliable with incorrect feedback condition (UNREL+IF). Although the level of trust in the REL+IF condition (M = 4.5, SD = 1.55) is higher than in the UNREL+IF condition (M = 4.19, SD = 1.17), the result of Mann-Whitney U test in this case did not show a statistically significant difference between the two conditions (*n* = 16, U = 142, *p* = 0.59). This implies that the effect of the reliable behavior of the robot on human trust is dominated by the effect incorrect feedback the robot provided.

## Discussion

Our focus in this work is to study the dynamics of human trust and its factors in collaboration with a robot, and to explore means that can be used by the robot in order to maintain and calibrate it should the need arise. This is an essential starting point to develop a computational model that can be implemented on the robot controller to make it aware of the trust level of its human partner and helps it adjust its behavior in accordance.

### Trust Dynamics and the Associated Factors

We empirically found that trust shows different dynamics depending on the direction of its evolvement. Its dynamics seems to be twice as strong while dissipating than while accumulating (see Figure 7). Our results correspond with previous theoretical work (Juvina et al., 2019) and empirically supports it (RQ1).

In the accumulation phase, four factors show a strong correlation with human trust, which are dependability, reliability (starting from the second step), predictability, and faith as can be observed in Table 3. The first step’s result shows the same three factors appear in the model of Rempel et al. (1985) and Muir (1994), and these factors continue to correlate with trust as it increases. Thus, the result of the first step agrees with the interpersonal trust model. However, our results disagree with the hierarchy of it, because in our case, the three factors appeared together in the first step of the interaction, whereas the factor of predictability should have dominated at the early stage of the interaction, then dependability, and faith afterward according to the model. Perceived reliability appears from the second step onward. The reason of its latency can be that the participants first needed to observe how the robot would handle a rusty screw before they judge its reliability.

Additionally, we could not find factors that strongly and consistently correlate with trust as it dissipates (see Table 4), which could be due to the small sample size used in this study. Only the factor adaptability appears twice at the end of the run. This suggests that this factor might play a strong role in the trust dissipation phase. Thus, the ability of the robot to adapt to the user’s preferences could contribute to maintaining the level of human trust in the collaboration. Although we did not identify clear factors in this direction, it shows evidence to a very interesting conclusion; that is, trust might have different factors in the dissipation phase than in accumulation one. Any method or instrument to measuring trust, be it subjective or objective, must take these characteristics of trust into account in order to provide reliable measurements. This has been overlooked by researchers since trust is mostly being measured with a post-hoc questionnaire once at the end of an experiment, with no guarantee that the questionnaire actually captures trust accurately enough. Although this finding does not fully answer the second research question (RQ2), it calls for researcher’s attention regarding the differences of these two phases. Accordingly, using a single instrument to measure trust will most likely fail to capture it. Therefore, multiple instruments are required to measure trust accurately.

The difference in the relevant trust factors during the accumulation and dissipation phases could be a reason why trust shows different dynamics in these two phases. These new findings about human trust dynamics need to be considered in future research on trust in order to capture it in real-time. This partially addresses the first research question (RQ1) and calls for further deeper investigations.

### Verbal Feedback Effect

In addition to the trust dynamic development, we also explored the potential effect of providing feedback by the robot on human trust and whether this can be used to preserve human trust and calibrate it when needed during the collaboration. We expected that correct verbal feedback will increase human trust even when the robot works reliably. However, the results of the study did not support this expectation and we found no statistically significant difference in the reported trust when correct feedback accompanies reliable behavior or not [see *Reliable Behavior With Feedback Correctness Changing (TC in G1)*]. Conversely, correct verbal feedback did increase the level of human trust when the robot fails to do its task [see *Unreliable Behavior With Feedback Correctness Changing (TC in G2)*]. Accordingly, the results imply that correct verbal feedback might be redundant in case the robot works as it should but essential if failures are expected. This does not comply with the findings of previous work of Desai et al. (2013), as feedback did not affect human trust in their study. Besides, correct feedback does have a positive impact on trust when the robot works unreliably because it works as a warning as most participants stated after the experiment which has been perceived by participants as a good behavior [see *Unreliable Behavior With Feedback Correctness Changing (TC in G2)*]. These results underline how important an assurance is when a failure is expected. It may be able to prevent trust of the human from draining beyond recovery.

Incorrect verbal feedback, on the other hand, did not have a strong impact on human trust if the robot acts unreliably. It seems to have influence only if the robot acts reliably [see *Reliable Behavior With Feedback Correctness Changing (TC in G1)* and *Unreliable Behavior With Feedback Correctness Changing (TC in G2)*].

Overall, the results show how sensitive trust is to feedback and suggest that feedback can actually be an effective way to calibrate trust. It seems that human trust is more sensitive to incorrect feedback than it is to correct feedback when the behavior is reliable, and it is more sensitive to correct feedback than incorrect feedback when the behavior is unreliable. The conclusion that emerges from that is interesting mainly for designers, since it implies that the system does not need to provide feedback unless a failure is expected. Our results contribute to existing the models put forward about these assurances, e.g., the trust cycle of Israelsen and Ahmed (2019), as our results shows that those assurances have a different impact on the level of human trust depending on other factors which might sometimes dominate over assurances (the reliability of the behavior in our case). This helps in answering our third research question (RQ3). Additionally, as verbal feedback affected trust in both directions, one can conclude that verbal feedback has the potential to actually calibrate human trust during the execution of a task.

Regarding our fourth research question (RQ4), the results of the analysis suggest that the use of correct feedback is effective only if an unreliable behavior is expected, and the incorrect feedback has an influence on human trust only if the behavior is reliable. Therefore, providing correct feedback might help in preserving human trust during the collaboration although it might be redundant in some cases. Additionally, providing incorrect feedback can help in lowering human trust when it gets too high.

Moreover, if we consider the levels of the used variables as Booleans (unreliable: false, reliable: true; incorrect feedback: false, correct feedback: true; trust not affected: false, trust affected: true), then the conclusion from analyzing the different conditions gets very interesting. It seems like human trust is affected (true) if only one of the variables (reliability and feedback) is true, and it is not affected (false) otherwise, which means that trust sensitivity (affected or not) is the result of the exclusive disjunction operation *XOR* between the used variables as shown in Table 5.

**TABLE 5:** Boolean logic relationship between reliability and feedback and their effect on human trust compared to the case where no feedback is provided.

Reliability		Feedback		Logic	Trust	
Reliable	(1)	Correct	(1)	XOR	Not affected	(0)
Reliable	(1)	Incorrect	(0)	XOR	Affected	(1)
Unreliable	(0)	Correct	(1)	XOR	Affected	(1)
Unreliable	(0)	Incorrect	(0)	XOR	Not affected	(0)

This helps also in answering the fourth research question (RQ4) and modeling human trust. To reduce human trust when it is too high, purposefully incorrect feedback might help bringing it back to the safe zone. On the other hand, if it is low because of failures, then correct feedback might help in preserving human trust, which makes the verbal feedback a valid means for trust calibration.

### Study Limitations

Although this study contributes toward better understanding on human trust in collaboration with a robot, its calibration, and the factors that might influence it, the study has its own limitations that can be addressed in future research. Firstly, the use of a mixed-reality environment had an influence on participant’s perception of safety. Most of the participants of our study felt safe during the collaboration with the virtual robot, which might have affected participant’s subjectively reported trust. Prior work (see *Environment Choice*), though, suggested that using virtual reality to study human-robot interaction is a valid option. Therefore, we believe that the results presented in this study provide reasonably valid contributions and we are currently planning a follow-up experiment with the same robot used in this study but in a real physical collaboration setting in order to make the environment closer to the real application.

Secondly, all of our participants were university students and employees, who have very limited (if any) practical experience with the disassembly processes and their complexity. Although the results provide us with better insights on human trust in collaboration with a robot, this limitation might have a strong impact on the external validity of the experiment.

Thirdly, the conclusions drawn regarding the factors that guide human trust in the collaboration setting (TD phase) were based mainly on correlational analysis, which does not necessarily imply causation. For better and more robust results, the causal relationship between the used factors and trust needs to be investigated in similar physical collaboration setting.

Finally, the sample size in the conditions of the TC phase, in which feedback and reliability influences on human trust were investigated, is relatively small to draw generalizable conclusions. This might also be the reason why we were not able to find any statistically significant differences between men and women in trust which does not conform with previous studies where women reported higher trust in Gallimore et al. (2019) and lower trust in Schuster et al. (2015) than men. A larger sample size would better represent the population allowing for the use of parametric tests and enhancing the power and generalizability of the results.

## Conclusion and Future Work

In this study, we explored multiple aspects of human trust toward a robotic team partner. Mainly, we were interested in understanding human trust evolvement over time when collaborating with a robot in a shared workspace and whether it can be calibrated by the robot to avoid inappropriate reliance. Essential to this is knowing the factors that could affect trust development. Therefore, we started by examining the relevant factors in a human-robot hand-in-hand collaboration setting. We differentiated between trust accumulation and dissipation phases by considering each one separately. We found four relevant factors that strongly correlate with trust in its accumulation phase. They are: dependability, reliability, predictability, and faith. However, none of the proposed factors correlated with trust in the dissipation phase. This can be due to the small sample size, but it points to a very interesting conclusion, which is that trust factors in its accumulation phase differ from those in its dissipation phase. Additional research is required to obtain better understanding on these factors and their effect on trust development. Further, we investigated the dynamics of trust in the aforementioned phases. We detected a stronger dynamic behavior in the dissipation phase compared to the accumulation one, which conforms with previous theoretical research. In addition to trust dynamics, we also investigated the impact of the verbal assurance provided by the robot on human trust in different conditions and whether it contributes to trust calibration. Our findings suggest that verbal feedback has the merit to influence human trust positively and negatively depending on its correctness which makes it a strong candidate to be deployed in order to calibrate trust of a human partner.

Although the study has some limitations, its findings meaningfully contribute to our knowledge about human trust in a robotic partner and provide insights to designers for a better collaboration quality.

One possibly important limitation was the use of a virtual robot, which might have strongly reduced participant’s perception of vulnerability as their perception of safety was mostly high. To approach this limitation, we are planning a similar experiment with a real physical robot.

Our future work will concentrate further on finding trust factors which is an initial step toward developing a reasonable quantitative model. The implementation of this model on the robot’s controller could make it aware of the human trust which allows it to adapt its behavior accordingly.

## Data Availability

The raw data supporting the conclusions of this article will be made available by the authors, without undue reservation.

## Footnotes

1
https://www.franka.de/

2
https://www.simzentrum.de/hermes/

3
http://www.electrocycling.de/en/welcome

4
https://www.umicore.com/

5
https://www.microsoft.com/en-us/hololens/

6
https://unity.com/
