# Corrigendum: Trust Dynamics and Verbal Assurances in Human Robot Physical Collaboration

**DOI:** 10.3389/frai.2021.761184

**Published:** 2021-10-01

**Authors:** Basel Alhaji, Michael Prilla, Andreas Rausch

**Affiliations:** ^1^ Simulation Science Center Clausthal-Göttingen, Clausthal University of Technology, Clausthal-Zellerfeld, Germany; ^2^ Institute for Informatics, Clausthal University of Technology, Clausthal-Zellerfeld, Germany; ^3^ Institute for Software and System Engineering, Clausthal University of Technology, Clausthal-Zellerfeld, Germany

**Keywords:** human-robot collaboration, trust dynamics, trust factors, trust calibration, human robot teamwork, verbal feedback

In the original article, there was a mistake in the figures and captions as published. The images were in the incorrect order and did not match the figure captions. The corrected Figures 1–7 appear below. Also the word “between” was duplicated in the caption of Figure 6, so we removed the duplicate.

**FIGURE 1:**
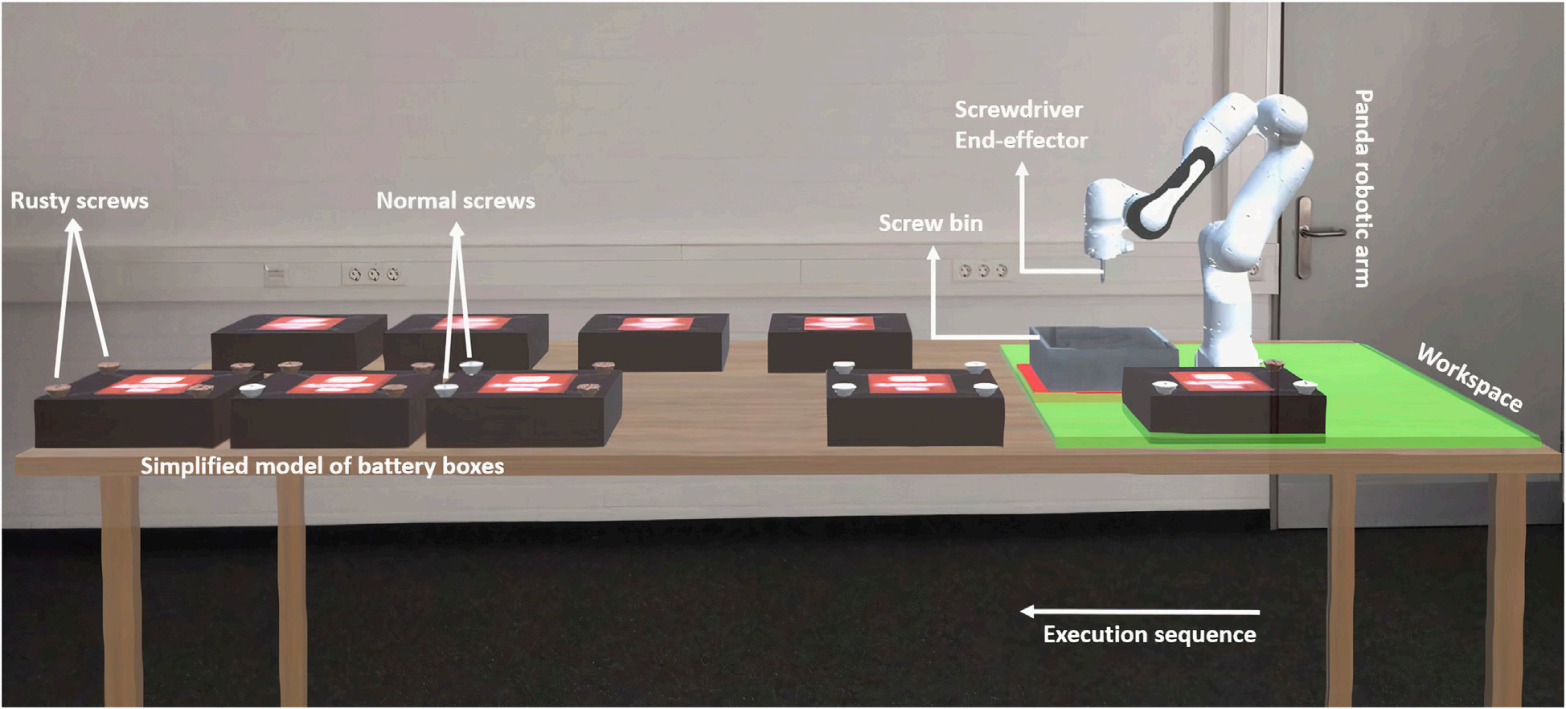
Experiment setup (from participant’s point of view). The green area shows the accessible workspace of the robot. There are two rows of batteries: on the front side, the batteries with screws are located; on the back side, some extra already disassembled batteries are located to create a more realistic scene.

**FIGURE 2:**
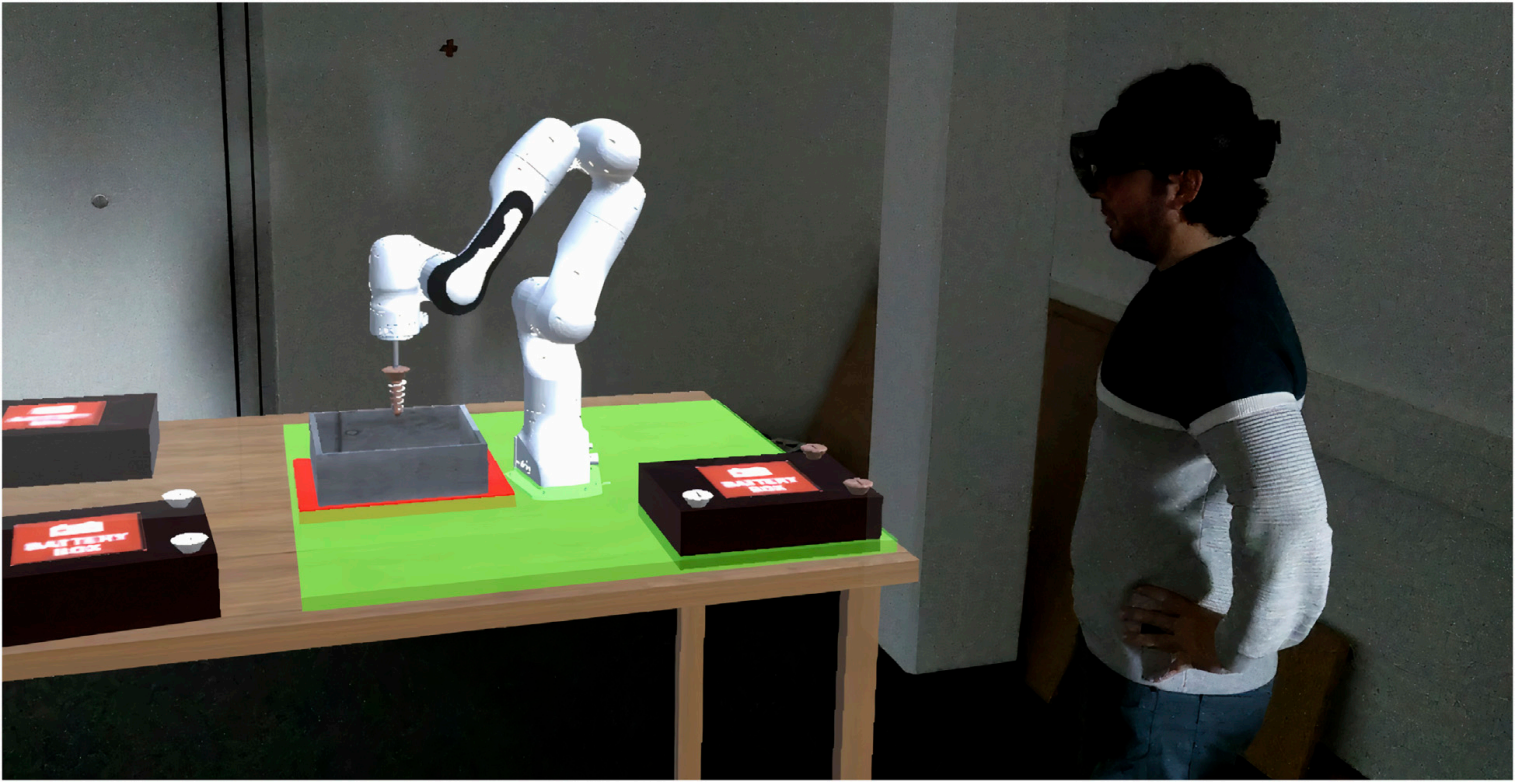
Third party point of view. A participant wears the HoloLens and commands the robot to loosen screws. It is allowed to locate the battery anywhere inside the green workspace except over the screw bin which is marked in red.

**FIGURE 3:**
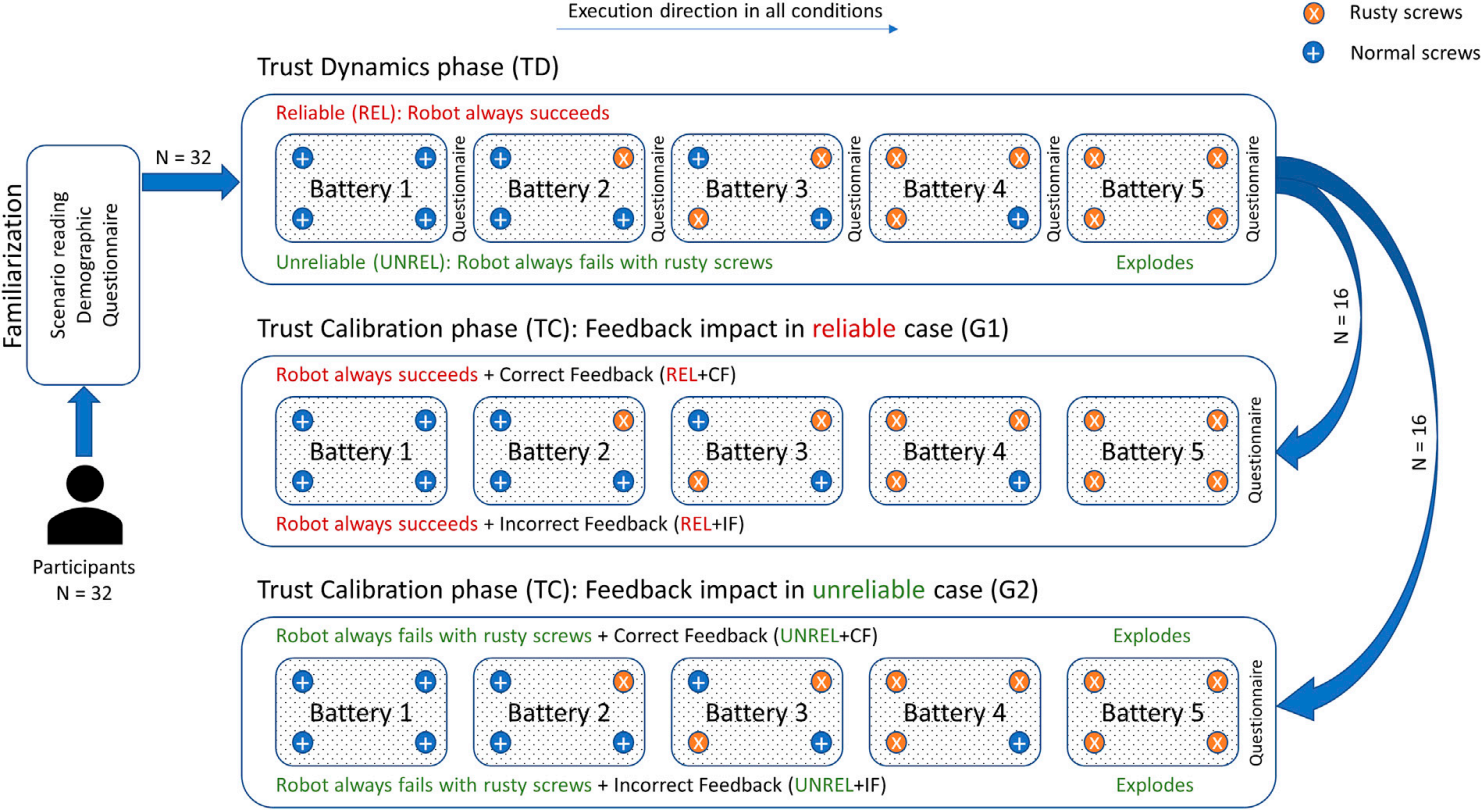
Experimental procedure. Participants start with TD phase and fill a questionnaire after each battery. They proceed with TC phase depending on the group, where only one questionnaire is administered. Batteries should be handled in their order. In TD phase, trust dynamics is studied. In the TC phase, the effect of verbal feedback on human trust is studied with different reliability levels.

**FIGURE 4:**
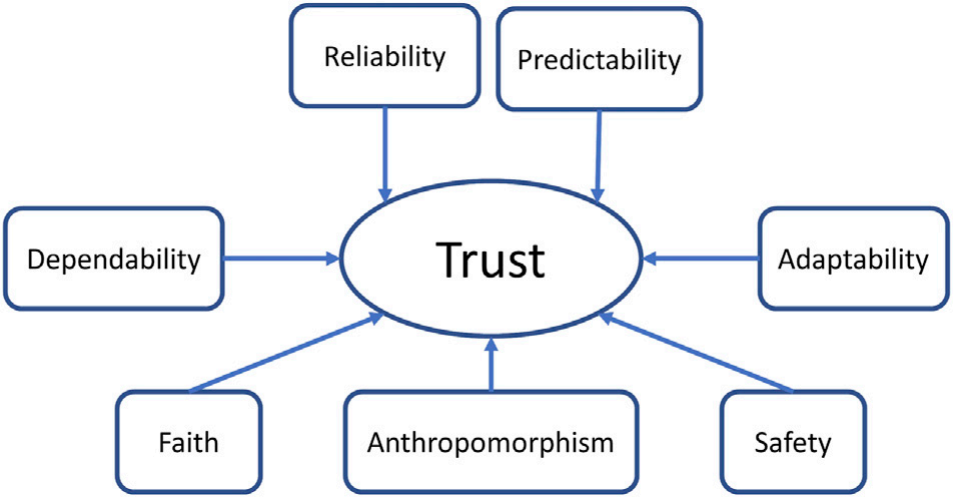
Potential trust factors included in this study.

**FIGURE 5:**
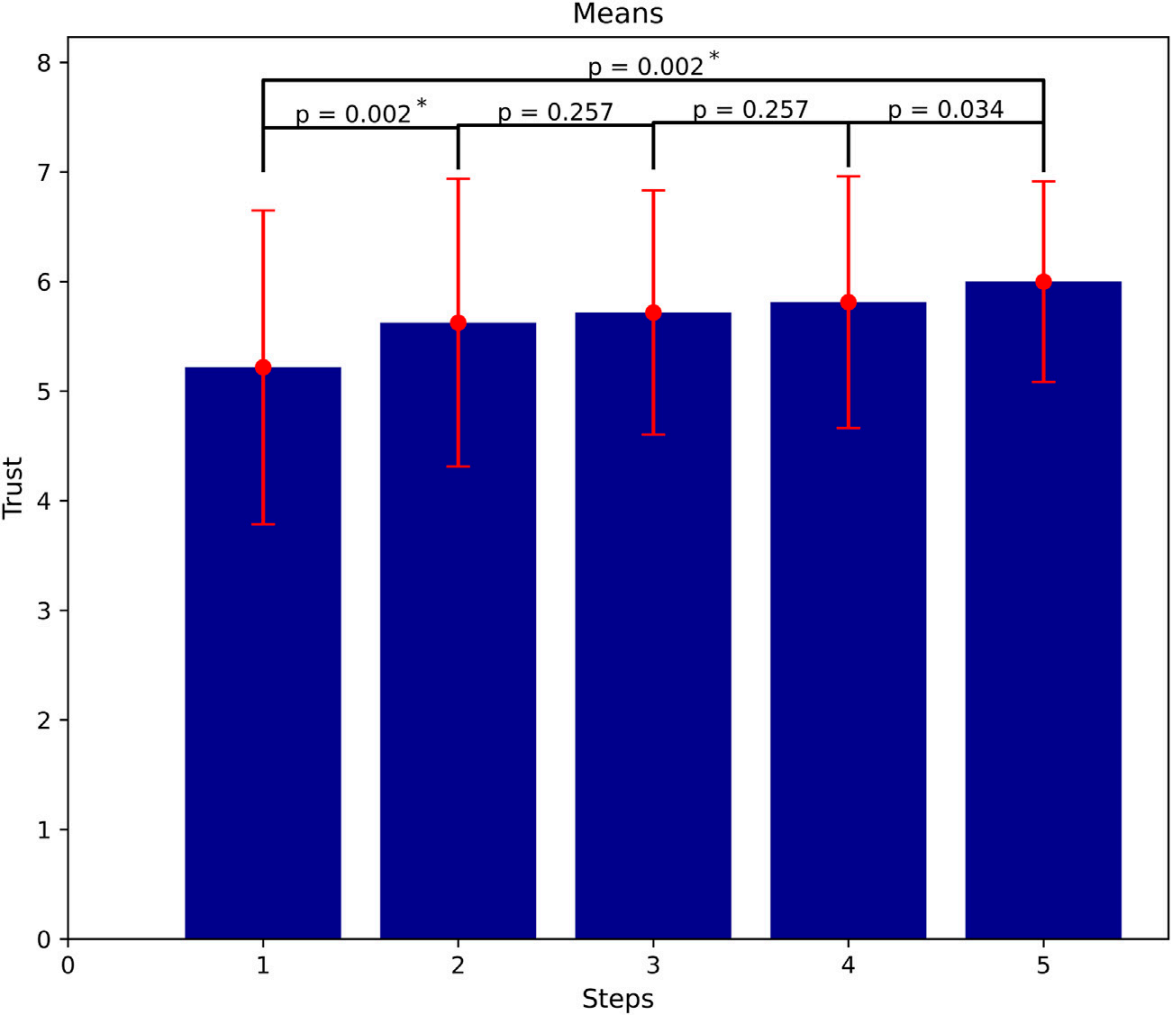
Trust accumulation as more successful interactions and task executions occur (means of trust at all steps). Significant gain of trust between the first and the last steps, but not in between them (α = 0.01).

**FIGURE 6:**
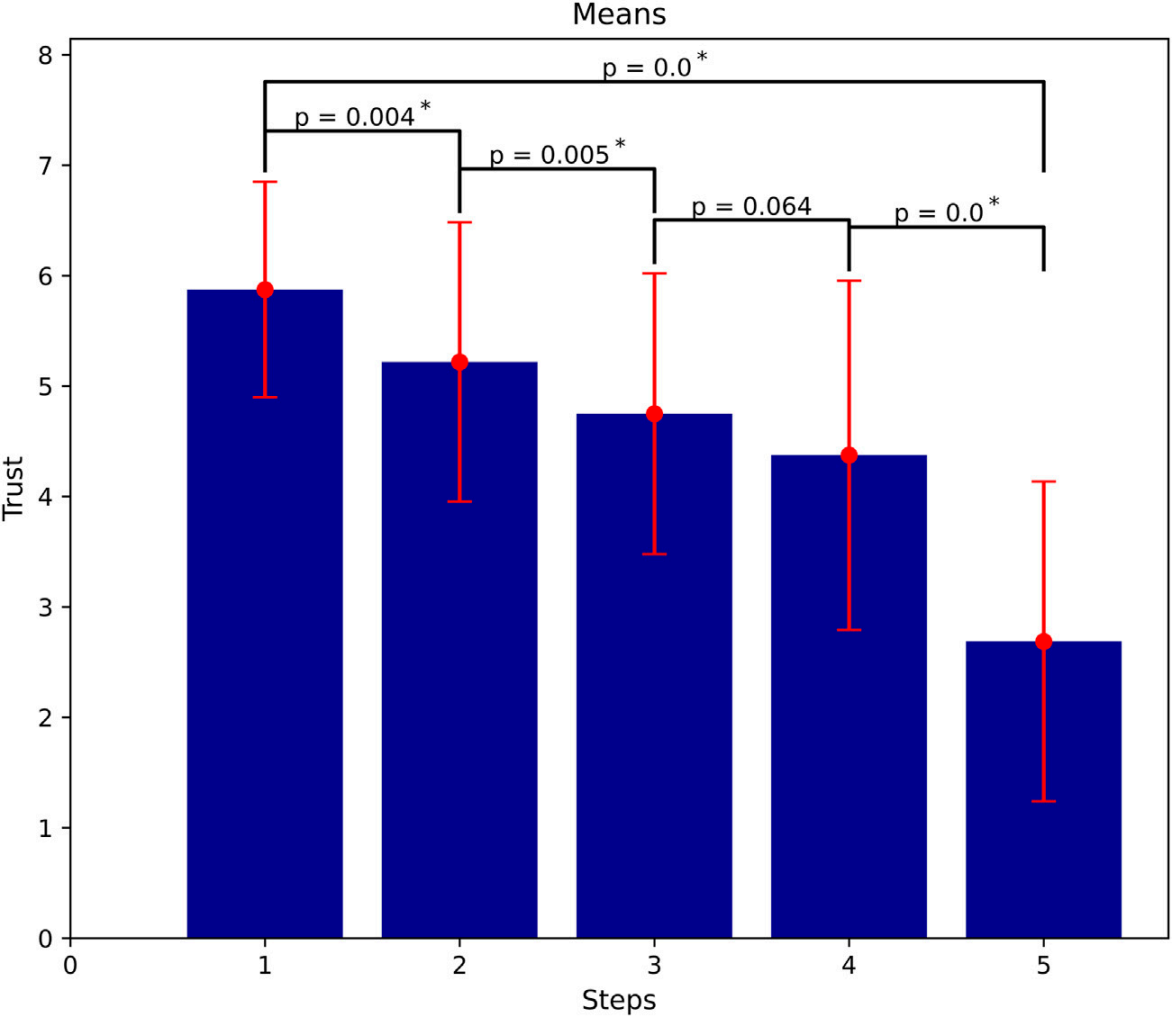
Trust dissipation as more failures occur (means of trust at all steps). Significant loss of trust mostly even in between steps (α = 0.01).

**FIGURE 7:**
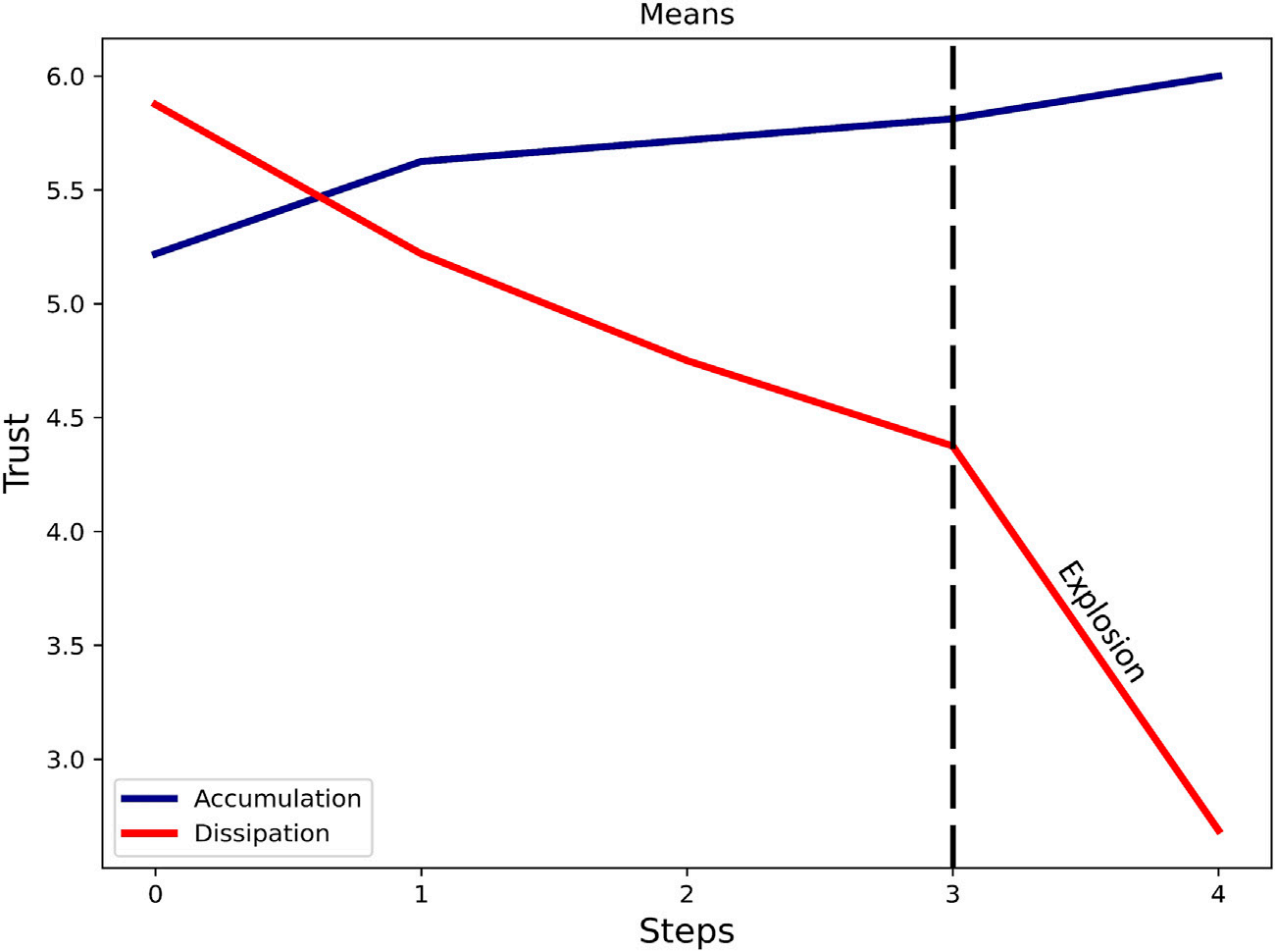
Dynamic trust development. Almost doubly stronger dynamics in dissipation compared to accumulation.

The authors apologize for this error and state that this does not change the scientific conclusions of the article in any way. The original article has been updated.

